# *Chlamydia caviae* in Swiss and Dutch Guinea Pigs—Occurrence and Genetic Diversity

**DOI:** 10.3390/pathogens10101230

**Published:** 2021-09-23

**Authors:** Silvia Ciuria, Michael S. M. Brouwer, Marende M. de Gier, Yvonne van Zeeland, Alex Bossers, Barbara Prähauser, Julia Schädler, Jean-Michel Hatt, Marloes Heijne, Nicole Borel

**Affiliations:** 1 Institute of Veterinary Pathology, Vetsuisse Faculty, University of Zurich, 8057 Zürich, Switzerland; silvia.ciuria@uzh.ch (S.C.); barbara.praehauser@uzh.ch (B.P.); 2 Center for Clinical Studies, Vetsuisse Faculty, University of Zurich, 8057 Zürich, Switzerland; 3 Department of Bacteriology, Host-Pathogen Interaction and Diagnostics Development, Wageningen Bioveterinary Research, 8221 RA Lelystad, The Netherlands; mike.brouwer@wur.nl (M.S.M.B.); alex.bossers@wur.nl (A.B.); marloes.heijne@wur.nl (M.H.); 4 Department of Clinical Sciences, Faculty of Veterinary Medicine, Utrecht University, 3584 CM Utrecht, The Netherlands; m.m.degier2@students.uu.nl (M.M.d.G.); y.r.a.vanzeeland@uu.nl (Y.v.Z.); 5 National Reference Centre for Poultry and Rabbit Diseases, Institute for Food Safety and Hygiene, Vetsuisse Faculty, University of Zurich, 8057 Zürich, Switzerland; julia.schaedler2@uzh.ch; 6 Clinic for Zoo Animals, Exotic Pets and Wildlife, Vetsuisse Faculty, University of Zurich, 8057 Zürich, Switzerland; jmhatt@vetclinics.uzh.ch

**Keywords:** *Chlamydia caviae*, *Chlamydia psittaci*, *Chlamydiaceae*, *Cavia porcellus*, *Oryctolagus cuniculus*, zoonotic potential, genetic diversity, ompA genotyping

## Abstract

*Chlamydia (C.) caviae* is a known pathogen in guinea pigs, causing conjunctivitis, respiratory infections and abortions. Recently, a *C. caviae*-induced zoonotic link was identified as the etiology of severe community-acquired pneumonia in humans. Here, 784 conjunctival and rectal swabs originating from 260 guinea pigs and 110 rabbits from 64 husbandries in Switzerland, as well as 200 composite conjunctival swabs originating from 878 guinea pigs from 37 husbandries in The Netherlands were examined by real-time PCR followed by conventional PCR and sequencing. *Chlamydiaceae* were detected in 2.3% (18/784) and 12.5% (25/200) of all Swiss and Dutch samples, respectively. An overall *C. caviae* occurrence was detected in 2.7% (7/260) and 8.9% (78/878) of all Swiss and Dutch guinea pigs, respectively. *OmpA* genotyping of 64 *C. caviae*-positive samples resulted in 33 sequences sharing 100% nucleotide identity with the strains isolated from the zoonotic transmission cases in The Netherlands. However, all *ompA* sequences of this study were distinct from the *C. caviae* GPIC reference strain. *C. caviae* was not detected in rabbits but *C. psittaci* genotype A was identified in guinea pigs and rabbits, raising concerns about the importance of these animal species as novel zoonotic sources for *C. psittaci*.

## 1. Introduction

The *Chlamydiaceae* family is composed of one single genus, *Chlamydia* (*C.*). This genus includes fourteen different species and several *Candidatus* species of Gram-negative, obligate intracellular bacteria with a biphasic life cycle [1]. *Chlamydiaceae* are globally distributed and possess the ability to infect over 400 different hosts, from wildlife to pets and humans, with some chlamydial species considered strictly host-specific, while others are zoonotic [2].

For *C. caviae*, the main host is the guinea pig (*Cavia porcellus*). It was first isolated by Murray in 1964 from the conjunctiva of an infected young laboratory guinea pig [3]. In this host, *C. caviae* invades mucosal epithelial cells, particularly of the conjunctiva, as well as the lungs, urinary bladder and genital organs [4]. Infections can range from asymptomatic to systemic, with clinical signs such as conjunctivitis (GPIC, guinea pig inclusion conjunctivitis), purulent ocular discharge, chemosis, rhinitis, pneumonia, ascending genital tract inflammation and abortions [1]. Transmission can occur through close contact, vertically and sexually, spreading quickly among animals housed together [5]. Although the clinical signs following *C. caviae* infection are well known, data on its prevalence is limited. Lutz-Wohlgroth et al. showed a 48% (59/123) prevalence for *C. caviae* in guinea pigs from Switzerland, with 81% (48/59) of those exhibiting clinical signs [6]. However, these numbers were based on a prevalence study across animals presented with ocular signs at the Ophthalmology Unit of the Department for small animals at the University of Zurich, hence leading to an estimated prevalence within a diseased (sub)population, therefore likely overestimating the actual prevalence of the pathogen. As a result, information regarding *C. caviae* prevalence in healthy guinea pig populations remains scarce, despite their importance as popular companion animals, especially for children.

Several *Chlamydia* species such as *C. abortus* and *C. psittaci* have a well-known zoonotic potential, resulting in severe clinical symptoms in humans, such as atypical pneumonia, abortion and septicemia [7]. For *C. caviae*, the zoonotic potential was only recently identified. First, Lutz-Wohlgroth et al. retrieved *C. caviae* in the conjunctival swab of an owner of a diseased guinea pig flock who suffered from mild serous ocular discharge [6]. More recently, three patients in The Netherlands were hospitalized with community-acquired pneumonia with two of them diagnosed with severe respiratory insufficiency requiring mechanical ventilation for several days [8]. *C. caviae* was the only pathogen detected in the bronchoalveolar fluid of these patients. All three patients reported owning guinea pigs at home or were in contact with them in a veterinary clinic. The guinea pigs likewise showed respiratory signs and conjunctivitis prior to their owners’ illness [8]. A fourth patient diagnosed with severe community-acquired pneumonia was *C. caviae*-positive in the bronchoalveolar fluid and feces [9]. This patient did not report any prior guinea pig contact, raising questions about the zoonotic transmission source. In one of the patients in the Ramakers et al. report, the direct transmission from a guinea pig to its owner was confirmed through tandem repeat analysis and sequencing of the outer membrane protein A (*ompA*) coding region as both isolates were identical [8]. In these analyses, the investigated molecular features of the zoonotic strain were different from the *C. caviae* strain GPIC (GenBank accession number: AE015925) but shared homology with two German *C. caviae* strains (DSM 27655 and 04DC41; with GenBank accession numbers KY777665.1 and KY777667, respectively), raising questions about the molecular epidemiology of *C. caviae* circulating in the European guinea pig population. 

Despite veterinary recommendations not to mix animal species, many husbandries host guinea pigs and rabbits in the same enclosures. This can lead to increased stress, behavioral problems, miscommunication and potential pathogen transmission between these two species [10,11]. In general, knowledge on chlamydial infections in rabbits is limited. Historically, Iversen et al. reported an experimental inoculation with *C. psittaci* strain M56 (originally isolated from muskrats (*Ondantra zibethicus*) and snowshoe hares (*Lepus americanus*) in 1961 [12]) in snowshoe hares, which led to a surprisingly high mortality (18 deaths of the 19 exposed lagomorphs) [13]. The infection was shown to be an acute and febrile illness with terminal clinical signs including opisthotonos, convulsions and hypoglycemia [13]. Additional research from Iversen et al. (1974, 1976) demonstrated that experimental infections using the same strain in cottontail rabbits (*Sylvilagus floridans*) and domestic rabbits (*Oryctolagus cuniculus*) likewise led to a febrile illness, but with greatly diminished lethality and, for the first time, with ocular lesions (i.e., anterior uveitis and conjunctivitis) [14,15]. Until Lutz-Wohlgroth et al. reported *C. caviae* in a conjunctival sample of one rabbit exhibiting mild ocular discharge, no further research was done on this topic [6]. Lately, Ni et al. showed a *Chlamydia* seroprevalence of 17.9% (143/800) in domestic rabbits in China, but the involved *Chlamydia* species was not determined [16].

To address these questions, we aimed to (i) investigate the prevalence of *C. caviae* in guinea pigs in Switzerland and in The Netherlands as well as its zoonotic potential, (ii) determine chlamydial occurrence in rabbits with or without contact to guinea pigs, (iii) perform *ompA* genotyping of *C. caviae*-positive samples to assess any strain diversity and (iv) determine further genetic markers for genotyping based on genomic comparisons between the reference strain GPIC, the Dutch zoonotic strain NL_Conj_Li and the German strain 04DC41.

## 2. Results

### 2.1. Chlamydiaceae and C. caviae Prevalence Data

Details of the *Chlamydiaceae*-positive husbandries in the Swiss and Dutch study are shown in Table 1.

The overall percentage of positive Swiss samples for *Chlamydiaceae* was 2.3% (18/784 samples) with a mean Ct value of 32.8. Of these, a total of 2.7% (15/553) positive swabs were collected from 14 guinea pigs across six husbandries and a total of 1.3% (3/231) positive swabs were collected from three rabbits originating from three different husbandries. Of the sampled husbandries, three were hosting only guinea pigs, one only rabbits and the remaining five hosting both animal species in the same enclosures. The 18 positive samples were identified in 1.1% (4/370) of the rectal, 2.6% (2/77) of the individual conjunctival and 3.5% (12/337) of the pooled conjunctival swabs (Table 2). Eight samples originating from seven *Chlamydiaceae*-positive guinea pigs across two different husbandries could be further identified as *C. caviae*, resulting in a total Swiss *C. caviae* positivity of 2.7% (7/260 guinea pigs). From one guinea pig (G55), a paired *C. caviae*-positive conjunctival and rectal swab was available. In six *Chlamydiaceae*-positive samples (G65C, G67C, G68C, G191R, G238C and R103C), the chlamydial species could not be further identified. A complete list of all Swiss swabs collected and their results are presented in Supplementary Table S1.

The overall proportion of Dutch samples positive for *Chlamydiaceae* was 12.5% (25/200 samples), with a mean Ct value of 28.2 (Table 2). The 25 samples originated from 12% of the guinea pig population (105/878) belonging to five breeders (13.5%). A sample was a composite swab from a range of two to six animals. Twenty-one of the 25 *Chlamydiaceae*-positive swabs tested positive in the VD4 *C. caviae ompA* PCR, of which eleven could be confirmed as *C. caviae* with subsequent sequencing. The eleven *C. caviae*-positive swabs originated from 5.7% (50/878) of the sampled guinea pigs, found among three breeders. As certain samples could not be successfully sequenced, all 25 *Chlamydiaceae*-positive samples were analyzed by means of the *C. caviae* complete *ompA* gene PCR, resulting in an additional eight samples sharing 100% nucleotide identity with *Chlamydia caviae* clone Conj_Li *ompA* (GenBank accession number: KY777661). Altogether, in 19/200 of the Dutch samples *C. caviae* was identified, originating from 8.9% (78/878) of all sampled guinea pigs, found among 8.1% (3/37) of breeders. Due to the limited amount of DNA and low chlamydial load (high Ct values), the remaining six *Chlamydiaceae*-positive samples (sample number 62, 67, 98, 138, 189 and 191) could not be further classified. A complete list of all Dutch swabs collected and their results are presented in Supplementary Table S2. 

### 2.2. Molecular Typing of C. caviae from the Swiss and Dutch Samples

**Figure 1:**
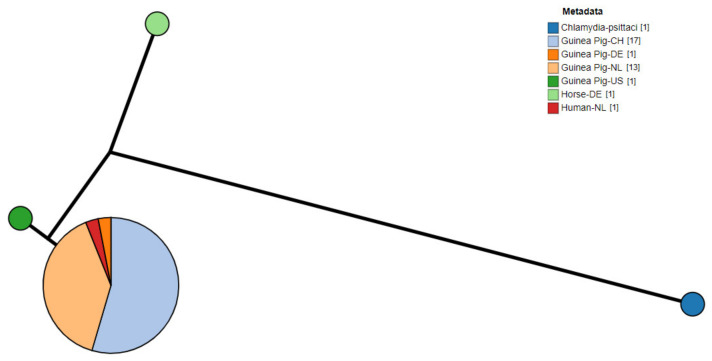
Phylogram of *C. caviae ompA* sequences from diverse sources. Metadata label shows the host and country of origin. *C. psittaci* was used as outgroup. (CH: Switzerland; NL: The Netherlands; DE: Germany; US: United States of America).

Of the positive samples (n = 64) amplified and sequenced, complete sequences for the *ompA* gene were successfully obtained from 33 samples, exclusively from guinea pigs. The Swiss samples of this study (n = 5, Supplementary Table S1) revealed 100% nucleotide identity with *Chlamydia caviae* clone NL_Conj_Li *ompA* gene (GenBank accession number: KY777661). The remaining 28 Dutch (n = 13, Supplementary Table S2) and previous Swiss samples (n = 15, [6]) had 100% nucleotide identity with the *C. caviae* strain isolated from the bronchoalveolar fluid of one of the patients in the Dutch zoonotic case [8] and its cultured clone (GenBank accession number: KY777661 and KY777669, respectively). All samples had a lower nucleotide identity (98.8%) with the *C. caviae* GPIC reference strain (GenBank accession number: AE015925.1). A phylogenetic tree based on the *ompA* sequences of different *C. caviae*-positive samples per country is displayed in Figure 1 [1,13,17]. Sequences were compared to the reference strain GPIC, the previously reported *C. caviae* 06G282 from a horse in Germany (GenBank accession number: GQ332575) and *ompA* from *C. psittaci* 6BC (GenBank accession number: CP002586.1) as an outgroup. While differences could be detected between GPIC and 06G282, *ompA* was identical in all other isolates reported here. 

### 2.3. Whole Genome Sequencing of C. caviae Isolates

As expected, the hybrid assembly of NL_Conj_Li resulted into one chromosomal contig and one plasmid contig and the chromosomal sequence of 04DC41 was present in nine contigs while the plasmid was present in one contig. The size of the chromosomes and plasmids is approximately 1.17 Mb and 7.5 kb, respectively, with 39% GC content and just under 1000 putative CDS, very similar to the previously published complete genome GPIC genome (Table 3). The average nucleotide identity of the complete isolates is >99%.

**Figure 2:**
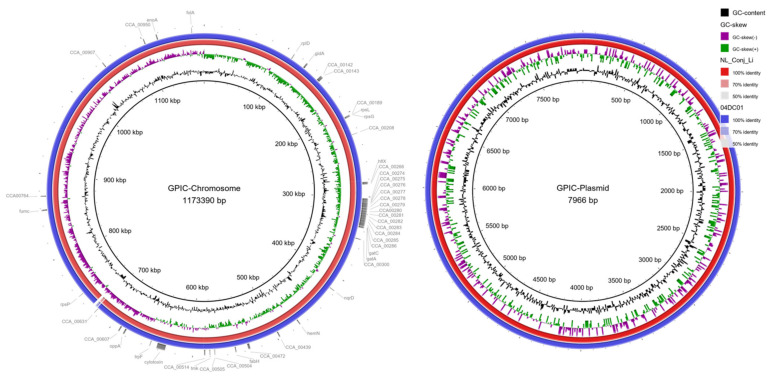
BRIG comparison of *C. caviae* genomes NL_Conj_Li and 04DC01 to reference strain GPIC. The left panel shows the comparison of the chromosome, the right panel is the comparison of the plasmid. From inside to outside, the rings represent the GC content, the GC skew and the percentage sequence identity to the reference genome. Putative gene targets that were compared for discrimination between NL_Conj_Li and 04DC01 are indicated in the outermost ring. One region is specifically divergent between NL_Conj_Li and 04DC01 compared to GPIC (see Figure 3, ~710 kbp).

**Figure 3:**
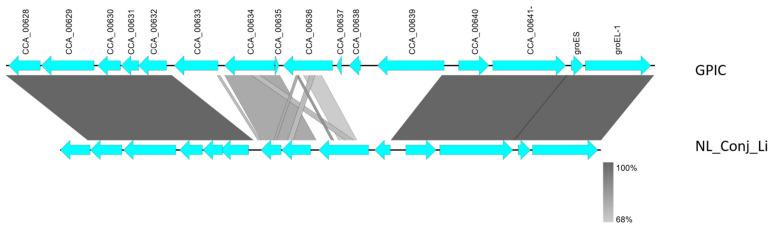
Detailed view of the genomic region divergent between NL_Conj_Li and 04DC01 compared to GPIC (see Figure 2). At close examination of this region, seven hypothetical genes in the GPIC chromosome are absent in NL_Conj_Li and 04DC01 and the region contains four predicted coding sequences. The function of these predicted genes is unknown, and the region is 100% identical between NL_Conj_Li and 04DC01.

Comparison of the complete genomes and plasmids using BLAST ring image generator (BRIG) underline the high degree of similarity between the genomes (Figure 2). The recently well characterized genes *sinC* and *incA* were compared between the three genomes as well as other putative divergent targets. While *sinC* and *incA* are 100% identical between NL_Conj_Li and 04DC41, the genes have 99.87% and 99.63% sequence identity respectively, compared to GPIC (Supplementary Table S3). One region is specifically divergent between NL_Conj_Li and 04DC41 compared to GPIC (Figure 2 and Figure 3). The region is 100% identical between NL_Conj_Li and 04DC41 and contains four predicted coding sequences. The region in GPIC encodes seven predicted coding sequences but no function can be predicted for any of the genes. No suitable genetic markers could be identified that would sufficiently discriminate between NL_Conj_Li and 04DC41 as target for further molecular screening.

### 2.4. Other Chlamydial Species in Guinea Pigs and Rabbits

In the Swiss samples, *C. psittaci* was detected in four samples from two guinea pigs and two rabbits (G161R, G184R, R59C_left and R68C) in the *C. psittaci* specific real-time PCR. Subsequent amplification and sequencing led to three samples (G161R, R59C_left and R68C) sharing at least 99.4% nucleotide identity with *C. psittaci* strain 6BC and 84/55 (GenBank accession number: NR_036864.2 and CP003790.1, respectively). Additional *C. psittaci*-specific *ompA* genotyping (1050 base pairs) of the four positive samples led to one additional *C. psittaci* isolate 84/55 (GenBank accession number: CP003790.1), originating from a guinea pig rectal sample (G184R). All *C. psittaci* confirmed samples shared the highest *ompA* similarity (97.3–99.6% nucleotide identity) with *C. psittaci* strain 84/55 (GenBank accession number CP003790.1), which had been isolated from a budgerigar and belongs to the *C. psittaci* genotype A [18]. *C. psittaci ompA* genotyping was unsuccessful in one sample (R59C_left). The four positive samples originated from four different husbandries, which kept both animal species in the same enclosures. None of the positive husbandries contained more than one *Chlamydia* species at the same time.

Three months after the first sampling, a second sampling of three *C. psittaci*-positive animals (two guinea pigs and one rabbit) ensued in negative results for all seven swabs (four conjunctival and three rectal swabs) by means of the 23S rRNA *Chlamydiaceae*-specific real-time PCR screening method (data not shown).

### 2.5. Clinical Signs in Chlamydiaceae-Positive Guinea Pigs and Rabbits

Clinical examination of all Swiss animals revealed a total of 24.9% (92/360) exhibiting either nasal discharge, ocular discharge or other ocular pathologies, all possible clinical signs related to a chlamydial infection. In detail, nasal discharge (serous, mucous, hemorrhagic or mucopurulent) was seen in 2.3% (6/260) and 15.5% (17/110) of the guinea pigs and rabbits, respectively. Furthermore, ocular discharge (serous, seromucous or mucopurulent) was noted in 7.7% (20/260) and 13.6% (15/110) of the guinea pigs and rabbits, respectively. Other ocular pathologies (i.e., lens opacification, subconjunctival deposition of fat, corneal lesions, accumulation of crusts and blindness) as well as typical *Chlamydia*-induced conjunctivitis signs (hyperemia, chemosis and inflammation of the conjunctiva) were reported in 21% (55/260) and 10.9% (12/110) of the guinea pigs and rabbits, respectively. Data from four deceased rabbits (4/35) was not available. Among the above-mentioned symptomatic animals, 4.4% (4/92) were positive for *C. caviae*, exhibiting either crusts bilaterally, subconjunctival fat deposition, serous ocular discharge or bilateral lens opacification, whereas 2.2% (2/92) were positive for *C. psittaci*, exhibiting an accumulation of crusts around the eyes, one also displaying mucous nasal discharge. Among the asymptomatic animals, 1.8% (5/278) displayed a positive *C. caviae* or *C. psittaci* result. *C. caviae*- or *C. psittaci*-positive symptomatic animals were found in 6.3% (4/64) of the sampled husbandries.

Clinical examination of all Dutch guinea pigs revealed a total of 3.4% (30/878) with clinical signs indicative for a chlamydial infection, distributed over 16 composite swabs. Ocular discharge (serous, seromucous, mucous or mucopurulent) was present in 56.7% (17/30) of the diseased guinea pigs, whereas nasal discharge (serous, mucous or purulent) was only observed in 20% (6/30) of the diseased guinea pigs. Ocular pathologies (i.e., corneal lesions, hypopyon, corneal vascularization) as well as typical *Chlamydia*-related conjunctivitis signs were recorded in 43.3% (13/30) of the diseased guinea pigs. Additionally, 10% (3/30) of the diseased guinea pigs showed respiratory sounds (i.e., rhonchi and stridor) during lung auscultation. Among the above-mentioned symptomatic animals, 20% (6/30) were positive for *C. caviae*, exhibiting either mucous or mucopurulent ocular discharge (5/6) or mucous nasal discharge (3/6). All six symptomatic animals were diagnosed *C. caviae*-positive by one composite swab and originated from one single Dutch breeder.

### 2.6. Symptoms Reported by Guinea Pig and Rabbit Owners

Reports from Swiss owners (n = 30) revealed that two owners of the six above-mentioned positive husbandries for *C. caviae* (n = 2) or *C. psittaci* (n = 4) suffered from respiratory symptoms in the previous year, specifically asthma and recurrent common colds, respectively. In addition, the latter mentioned owning cats with outdoor access which had a positive confirmed *Chlamydiaceae* result (*Chlamydia felis*) in the months prior. Reports from owners (n = 34) of the deceased rabbits were not available. 

Reports from the Dutch breeders (n = 37) revealed that none of the owners of the *C. caviae* positive husbandries (n = 3) suffered of any respiratory signs or pneumonia in the last five years.

## 3. Discussion

### 3.1. Prevalence Study in Swiss and Dutch Guinea Pigs

The present study detected an overall *C. caviae* prevalence of 2.7% (7/260) and 8.9% (78/878) in guinea pigs and of 6.6% (2/30) and 8.1% (3/37) husbandries in Switzerland and in The Netherlands, respectively. Both prevalence rates are considerably lower than the only available *C. caviae* occurrence data of 48% (59/123) previously reported in Switzerland [6]. In contrast to our study, Lutz-Wohlgroth et al. sampled predominantly ill guinea pigs that presented to the veterinary hospital in Zurich, with either clinical signs of ocular, genital or respiratory disease or a reduced general condition [6]. Half of the animals in the latter study originated from one breeder who kept the guinea pigs under suboptimal housing and hygiene conditions. We could speculate that the *C. caviae* prevalence reported at that time was high due to a chlamydiosis outbreak in the latter husbandry, and therefore was not representative of general *C. caviae* prevalence in Switzerland. In our study, randomly selected husbandries were sampled, with none of them reporting any major health issues in their animals, therefore the sample ought to better represent the *C. caviae* prevalence in the general guinea pig population.

In general, the prevalence at husbandry level in our study was low, but the prevalence within husbandries differed. This could be due to differences in husbandries (i.e., number of animals per m^2^, feeding and living conditions) and animal welfare. More than half of the *C. caviae*-positive samples (11/19) were detected in the husbandry of one Dutch trader (a business receiving different animal species and distributing them to pet shops/private owners), which may indicate that *C. caviae* can spread fast within one husbandry, as was also observed in the similar chlamydiosis outbreak in the Lutz-Wohlgroth et al. study [6]. Furthermore, the overall prevalence appeared to be lower in the Swiss study, which might be the result of smaller husbandries, the largest owning 50 guinea pigs (with a mean of 12 guinea pigs per husbandry), while the sampled Dutch husbandries kept up to 300 guinea pigs (with a mean of 39 guinea pigs per husbandry). It has been mathematically proven that a higher animal density and a direct mode of pathogen transmission can lead to increased pathogen spread [17,19]. However, the data between the Dutch and Swiss study cannot be easily compared due to the variation in husbandry types and the different sampling strategies used (composite swabs versus individual swabs).

### 3.2. Prevalence Study in Swiss Pet Rabbits

We did not detect *C. caviae* in rabbits with close contact to guinea pigs or in deceased rabbits, although *C. caviae* was previously detected in the conjunctival swab of a rabbit and a cat living in the same husbandry as a *C. caviae*-infected guinea pig flock [6]. The *C. caviae*-positive rabbit in the latter study displayed mild serous ocular discharge [6]. Ni et al. reported a *Chlamydia* seroprevalence of 17.9% (143/800) in pet rabbits in China, without determining which *Chlamydia* species was involved or if any clinical signs could be observed [16]. In our study, the *Chlamydia* occurrence in pet rabbits was 1.8% (2/110) and only *C. psittaci* genotype A was identified, but serology was not performed, making direct comparisons between studies difficult.

### 3.3. C. caviae Sequencing

In our study, all *ompA* gene sequences retrieved from Dutch and Swiss samples and the previous Swiss study [6] shared 100% nucleotide identity with the strains KY777661 and KY777669, isolated by Ramakers et al. [8]. Genotyping of the *ompA* gene is frequently used in *Chlamydia*, although resolution is limited and evolution might be divergent from other parts of the genome [20,21,22]. However, further characterization of *C. caviae* NL_Conj_Li and 04DC41 did not reveal any suitable genetic markers that would sufficiently discriminate both strains, while they were isolated at different timepoints (2014 versus 2004) and at different locations (Netherlands versus Germany). These results might indicate, that one *C. caviae* strain is currently circulating in European guinea pigs, which is different from the reference strain GPIC.

Comparisons of previously described regions of variability and potential virulence factors including the MLST genes, *incA*, *sinC*, *ompA*, the cytotoxin polymorphic outer membrane proteins, as well as the plasmid revealed differences with the reference genome GPIC but only a small number of SNPs between the sequenced isolates NL_Conj_Li and 04DC41. Further epidemiological studies on a global scale, including the typing of *C. caviae ompA* gene in other countries, are necessary to explore the *C. caviae* strain diversity worldwide. Such studies would require isolation and culturing of the pathogen to ensure that a single pathogen is sequenced, and that enough DNA can be isolated, which is specifically challenging for Oxford Nanopore sequencing due to the high amount of input DNA.

### 3.4. Clinical Signs in Chlamydia-Positive Animals

Typical clinical signs for a chlamydial infection in guinea pigs (i.e., conjunctivitis, ocular and nasal discharge, pneumonia, abortions and vaginal discharge) were present in 81.4% (48/59) of the *C. caviae*-positive animals of the previous Swiss study [6], whereas we only observed 11.8% (10/85) *C. caviae*-positive guinea pigs suffering from any clinical signs. As the majority of the infected animals in our study were asymptomatic, a persistent infection within a herd might go unnoticed, unless all animals (including asymptomatic ones) are tested. All positive symptomatic and asymptomatic animals should be treated with antibiotics (tetracycline) in order to reduce the spreading of the pathogen and therefore also lowering the zoonotic risk to the owners. In our field study setting, no complete ophthalmologic examination was conducted and additional typical clinical signs such as abortions as well as vaginal discharge were not included in our questionnaires (considering possible recall biases from the owners and lack of records for disease history in their animals). Therefore, it cannot be completely ruled out that more *C. caviae*-positive guinea pigs exhibited ocular or genital tract infection signs. However, a correlation between ocular clinical signs and *C. caviae* positivity was not observed in this study.

### 3.5. C. psittaci in Guinea Pigs and Rabbits—A New Zoonotic Risk?

Current veterinary advice recommends separation of guinea pigs from rabbits in order to minimize the potential pathogen transmission between these two species [11]. Pathogens such as *Bordetella bronchiseptica* and dermatophytes (i.e., *Trichophyton mentagrophytes* and *T. benhamiae*) are capable of infecting either species [11,23]. In our study, none of the husbandries had positive *C. caviae* or *C. psittaci* results in both animal species simultaneously. However, due to the intermittent shedding of *Chlamydia*, it might be possible that additional positive animals remained unnoticed.

Despite its broad host range, *C. psittaci* predominantly causes chlamydiosis in different avian species. Clinical signs range from asymptomatic to nasal and ocular discharge, air sacculitis, pneumonia, enteritis, hepatitis and chronic infections, the latter leading to intermittent shedding of the bacterium [24]. Avian chlamydiosis caused by *C. psittaci* is a notifiable disease in many countries (including The Netherlands and Switzerland). Reports on *C. psittaci* in cats, dogs, experimentally infected cattle and horses reflect its ability to induce ocular and respiratory signs as well as reproductive losses in other animal species [25,26,27,28]. Nonetheless, cases of clinical diseases in non-avian hosts are generally an exempt [29].

The only study on *C. psittaci* infections in domestic rabbits dates back to 1974, when substantial lethality and ocular pathologies (i.e., anterior uveitis and conjunctivitis) were reported in experimental infections [14]. However, these lagomorphs were infected with high doses of the *C. psittaci* M56 strain (isolated from muskrats and snowshoe hares during a die-off event some years previously [12]), while all positive animals in our study harbored the *C. psittaci* genotype A. Previously, the *C. psittaci* strain C6/98, belonging to genotype A (GenBank accession number: NZ_KE359921-NZ_KE360062) (data unpublished) had been isolated from a conjunctival swab of a domestic rabbit from Germany in 1998 (personal communication, K. Sachse). In our study, the two *C. psittaci*-positive guinea pigs showed no clinical signs, whereas the two positive rabbits had crusts around their eyes, one of them also displaying mucous nasal discharge but no conjunctivitis. At this point, the role of *C. psittaci* as a pathogen as well as the shedding frequency and duration of the agent in both animal species remain unresolved. Further investigations are thus needed to determine the prevalence, pathogenesis and presence of clinical signs in these animal species. Isolation and culture of *C. psittaci* strains from guinea pigs and/or rabbits will clarify their viability and infectivity and will help assess their potential danger for human transmission.

Due to the unexpected detection of the zoonotic chlamydial species *C. psittaci* in guinea pigs and rabbits in Switzerland as well as the high motivation of the corresponding owners for follow-up testing, a second sampling of the two positive guinea pigs and of one of the two rabbits was performed. All three animals were negative in the conjunctival and rectal swab samples by the 23S *Chlamydiaceae* screening method (data not shown). This could be attributed to (i) possible contamination of the conjunctivae and/or gastrointestinal tract at the time of the first sampling; (ii) potential chlamydial intermittent shedding with no detection at the second sampling; (iii) elimination of the agent by the host immune system or (iv) suboptimal sampling procedure performed by the owners with insufficient pressure applied, causing the flocked swabs to miss the infected cells. Still, further investigations of the *Chlamydia*-positive husbandries would be of interest to clarify the veterinary and potential public health concern arising from such *C. psittaci* infections.

### 3.6. Zoonotic Potential of C. caviae and C. psittaci

Except one individual suffering from asthma, owners of *C. caviae*-positive animals in our study did not report any respiratory discomfort in the years prior. None of the owners in Switzerland reported any signs of conjunctivitis. Therefore, our study did not provide further insight into the zoonotic potential of *C. caviae* as previously recognized [6,8,9]. Furthermore, we could not prove any zoonotic risks related to the presence of *C. psittaci* in guinea pigs and rabbits. In our study, one owner of a *C. psittaci*-positive guinea pig suffered from recurrent common colds, but due to ethical regulations, we were not allowed to take samples from the owners. Non-avian *C. psittaci* strains are considered to have a lower zoonotic potential, since most of the reported human psittacosis cases could be traced back to contact with avian species [29,30]. Additionally, *C. psittaci* strains isolated directly from mammals displayed significantly reduced infectivity and organ dissemination in an *in vivo* chicken embryo model when compared to the *C. psittaci* strains isolated directly from avian species [31]. However, in 2014, a cluster of psittacosis cases was reported in Australia when five veterinary students and university staff members suffered from fever, fatigue and pneumonia after contact with the infected fetal membranes of a mare with placentitis [32]. The abnormal fetal membranes were found to harbor the *C. psittaci* isolate 6BC belonging to the genotype A [33].

Although the present data and the absence of severe clinical signs in the guinea pigs, rabbits and owners do not warrant urgent and large-scale surveys in humans, either in Switzerland or in The Netherlands, the zoonotic potential of both chlamydial species should not be underestimated. It is therefore recommended to further characterize and type positive *Chlamydiaceae* results in symptomatic animals in order to confirm the presence or absence of possible zoonotic strains. In addition, we recommend that vulnerable populations such as children, immunosuppressed adults/owners and personnel in the veterinary field be informed about the zoonotic risks and remain cautious, particularly when animals present typical *Chlamydia*-related clinical signs.

## 4. Materials and Methods

### 4.1. Sampling of Swiss Guinea Pigs and Rabbits and Swab Preparation

Between August 2019 and March 2021, a total of 784 swabs were collected from 370 pet guinea pigs and rabbits from different geographical locations in Switzerland. In total, 260 guinea pigs from 29 different husbandries and 75 rabbits from 22 husbandries in 12 different Swiss cantons were sampled, some of these husbandries (n = 30) keeping both species in the same enclosure. Additionally, 35 deceased pet rabbits which were investigated by the National Reference Center for Poultry and Rabbit Diseases (NRGK) for cause of death were added to the sample set. These 35 additional rabbits originated from 34 different husbandries in 11 different Swiss cantons. Husbandries (n = 64) included breeders (n = 6), zoos and community centers (n = 2), pet shops (n = 2) and private owners (n = 54). The sample set included a pooled conjunctival swab (n = 337) and a rectal swab (n = 370) from each animal. If the animal showed any signs of an ocular disease (i.e., ocular discharge or ocular pathologies), conjunctival swabs from both eyes were taken individually (n = 77), leading to a total number of 784 swabs (Table 4). Dry small-scale flocked swabs (FLOQSwabs Copan Flock Technologies, Brescia, Italy) were used for sampling and stored at −20 °C until DNA extraction. Additionally, age, sex, breed, health status, clinical signs, living conditions and geographic origin of the animals, as well as health status and clinical signs of the owners were noted. The study was conducted in strict compliance with the Animal Welfare Act of Switzerland. It was approved by the Cantonal Veterinary Office of Zurich (approval number ZH129/2019, 31548).

For the DNA extraction of the Swiss swab samples, a commercially available kit (Maxwell® 16 DNA Purification, Buccal Swab/LEV, #AS1295, Promega, Fitchburg, WI, USA) was used according to the manufacturer’s instructions. Using the Maxwell® 16 machine (Promega AG, Dübendorf, Switzerland) for the DNA extraction, a final elution volume of 50 µL was obtained.

### 4.2. Sampling of Dutch Guinea Pigs and Swab Preparation

Between October and November 2019, a total of 878 guinea pigs from 37 different breeders were sampled in eight different provinces in The Netherlands and one in Belgium (Table 4). The 37 husbandries included show breeders (n = 20), non-show breeders (n = 12) and petting farms (n = 5). Per husbandry, multiple composite swab samples were taken (Aluminum swab (sterile), Medical direct, The Netherlands), one composite swab including the sampling of one to six guinea pigs. This resulted in 200 composite swab samples, originating either from asymptomatic (n = 184) or symptomatic (n = 16) guinea pigs (Supplementary Table S2). One single-sided conjunctival sample was collected per guinea pig. Swabs were stored at −20 °C until DNA extraction. All guinea pigs were assessed for their overall health status prior to sample collection, with a brief clinical examination of the respiratory system, including the nose and eyes. Additionally, age and sex of the symptomatic guinea pigs, size and disinfection methods of the enclosures as well as health status and clinical signs of the owners were noted. The study was conducted in strict compliance with the Animal Act 2011 in The Netherlands. In accordance with the national regulations on animal experimentation, no ethical approval for the sampling was needed.

DNA extraction was performed with a NucliSENS easyMAG (Biomerieux, Zaltbommel, The Netherlands). Swabs were suspended in 1.5 mL Tryptose Phosphate 2.95% w/v with 52 mg/L gentamicin (BM 330, WBVR, Lelystad, The Netherlands) and thoroughly vortexed. From this suspension, 500 μL was added to 2 mL NucliSENS lysis buffer for off-board lysis. After at least one hour of incubation at room temperature, the lysis buffer was added to 80 μL of silica and extracted according to the manufacturer’s instructions for specific protocol B. Within this protocol, an optimized washing protocol was used with extra and longer washing steps. The final elution volume was 100 μL.

### 4.3. Chlamydiaceae Screening of Swiss and Dutch Samples

All extracted samples (n = 984) were first screened using the 23S rRNA *Chlamydiaceae*-specific real-time PCR, resulting in an amplicon of 111 base pairs and using primers Ch23S-F, Ch23S-R and probe Ch23S-p (Supplementary Table S4) [34]. Internal positive controls included enhanced green fluorescent protein (eGFP, [35]) for the Swiss samples and the ChMIX3IPC-template for the Dutch samples (Table 5). All Swiss samples were tested in duplicate. In each run, molecular grade water was used as negative control. For the Swiss samples, a sevenfold dilution of *Chlamydia abortus* DNA with a defined number of DNA copies was added in each run and used as positive control and standard curve. The threshold value for *Chlamydiaceae* was set at 0.1 and a sample was considered positive when both duplicates showed a mean cycle threshold (Ct value) < 38. If the duplicates showed a higher Ct value or inhibited amplification, they were repeated in duplicate and in a tenfold dilution. Samples repeatedly showing a slightly higher value than 38 (i.e., Ct values of 40) were considered questionably positive and submitted for further typing.

For the Dutch samples, a dilution series of three *Chlamydia psittaci* DNA samples were used as positive control [36]. Dutch samples were submitted for further typing when Ct values were <40.

### 4.4. Typing of Chlamydiaceae-Positive Samples 

**Figure 4:**
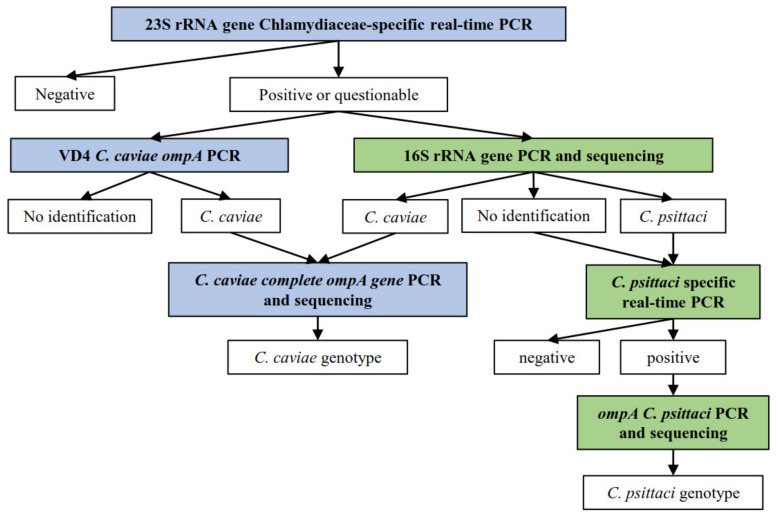
Decision tree for the sample processing, according to the different methods applied in each country, in which 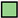 displays the methods applied only for the Swiss sample set, whereas 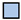 displays the methods applied on both Dutch and Swiss sample set. 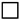 depicts the results obtained for each method.

Positive samples by the *Chlamydiaceae* screening PCR were further investigated according to the decision tree displayed in Figure 4. All primers and probes used in the further PCR tests are listed in Table 5. All Swiss primers and probes were purchased from Microsynth (Balgach, Switzerland). All Dutch primers and probes were purchased from Eurogentec, Life Technologies Europe, Genscript, Biolegio and Integrated DNA Technologies. All reaction mix concentrations and cycling protocols are summarized in Supplementary Table S4.

All positive or questionable Swiss samples in the 23S rRNA *Chlamydiaceae*-specific real-time PCR were further investigated by the 16S rRNA conventional PCR [37]. A short fragment of 278 base pairs in the conserved 16S gene was targeted. Primers 16S IGF and 16S IGR were used. *C. suis* DNA and molecular grade water were added to each run as positive and negative controls, respectively. Cycling was performed on the Thermocycler Biometra TProfessional Trio (Labgene Scientific, Switzerland) and each PCR product was confirmed for correct product length by gel electrophoresis on a 1.5% agarose gel plus GelRed® Nucleid Acid Stain (Biotium, California, USA), which ran for 45 min at 100 V and 400 mA. A 100 bp GeneRuler DNA Ladder (Thermo Scientific, Vantaa, Finland) was loaded and each sample dyed with 6× DNA Loading Dye (Thermo Scientific, Vantaa, Finland). PCR products were then viewed using BioDoc-It® 220 Imaging System (UVP, Cambridge, United Kingdom) and positive templates in either length were purified using the GeneJET PCR Purification Kit (Thermo Fisher Scientific, Waltham, MA, USA) following manufacturer’s instructions. Purified samples were Sanger-sequenced by Microsynth. Sequences were then assembled and evaluated using Geneious Software (Version 2021.0.1, available online: https://www.geneious.com, accessed from March to May 2021) and compared against sequences in the NCBI database using the BLASTn tool (Available online: https://blast.ncbi.nlm.nih.gov/, accessed from March to May 2021).

All Dutch samples with Ct values < 40 and inconclusive Swiss samples in the *Chlamydiaceae* 23S rRNA real-time PCR were further analyzed by means of a conventional PCR targeting the variable domain 4 of the *C. caviae ompA* gene [38]. Primers CCVDF and CCVDR were used to target a 130 base pairs amplicon. Reactions ran on the Stratagene MX3005P qPCR system (Applied Biosystems, Waltham, MA, USA). PCR products of samples with a melting curve with a Tm between 79 °C and 83 °C were forwarded for Sanger-sequencing by Baseclear BV (Leiden, The Netherlands). Sequences were then aligned using the Sequencer 4.10.1 and compared against available *C. caviae* sequences from the NCBI database.

### 4.5. Typing of C. psittaci-Positive Swiss Samples 

To assess all *Chlamydiaceae*-positive Swiss samples for the zoonotic chlamydial species *C. psittaci*, a *C. psittaci-specific* qPCR was performed according to Pantchev et al. [39]. The target product consists of an amplicon of 76 base pairs and the qPCR includes an internal positive control (eGFP) [35]. The PCR consisted of primers CppsOMP1_For, CppsOMP1_Rev and probe CppsOMP1 as well as eGFP_For, eGFP_Rev and probe eGFP_probe. All samples ran in duplicate on the QuantStudio 5 and Applied Biosystems™ 7500 Real-Time PCR System (Thermo Fisher Scientific, Switzerland). Synthesized oligonucleotide of the *ompA* gene of *C. psittaci* and molecular grade water were added in each run as positive and negative controls, respectively. Samples were considered positive when a Ct value was generated.

All *C. psittaci*-positive samples were further investigated by using a *C. psittaci ompA* PCR as described by Sachse et al. [40], which targets a 1050 base pair amplicon. Primers ompA F (CTU) and ompA rev were used and the PCR ran on the Thermocycler Biometra TProfessional Trio (Labgene Scientific, Châtel-Saint-Denis, Switzerland). Additionally, a positive (*C. psittaci* T49/90) and a negative control (molecular grade water) were added in each run. Gel electrophoresis and sequencing was performed as previously described.

### 4.6. OmpA Typing of C. caviae-Positive Swiss and Dutch Samples

All confirmed *C. caviae* positive Swiss and Dutch samples in this study (n = 27) were evaluated by a *C. caviae*-specific PCR based on the complete *ompA* gene, as described by Ramakers et al. [8]. Additionally, 37 positive conjunctival samples originating from the previous Swiss study on *C. caviae* prevalence in guinea pigs were added to the sample set [6]. All *C. caviae* complete *ompA* sequences were assembled and analyzed using Sequencher 4.10.1. Sequences were then compared against the sequence of the *C. caviae* GPIC reference strain and NL_Conj_Li (Genbank accession numbers: AE015925.1, KY777661.1). A maximum-likelihood tree of *ompA* sequences was calculated using MEGA 7.0.26 and visualized using Grapetree (1.5.0).

### 4.7. Whole Genome Sequencing

*C. caviae* isolates NL_Conj_Li and 04DC41 were cultured using Buffalo Green Monkey (BGM) cells as previously described [41]. *C. caviae* NL_Conj_Li was originally isolated from a conjunctival swab of a Dutch guinea pig that was related to the zoonotic case in 2014 [8]. *C. caviae* 04DC41 was originally isolated from a lung tissue of a guinea pig in Germany in 2014 (personal communication, C. Schnee). Genomic DNA was extracted using the DNeasy Blood and Tissue kit (Qiagen GmbH, Hilden, Germany) according to the manufacturer’s protocol, but with an additional incubation step of 10 min at 70 °C after addition of buffer AL. Whole genome sequencing of the Dutch zoonotic isolate NL_Conj_Li was performed on DNA of the third passage with Illumina sequencing. The passage number of the German isolate 04DC41 was unknown. Genomic DNA of isolates NL_Conj_Li and 04DC41 was prepared for sequencing on the Illumina MiSeq PE250 platform using the TruSeq kit (Illumina, San Diego, CA, USA) according to the manufacturer’s protocol. Illumina reads were adapter-filtered and quality-filtered using BBDuk (BBMap suite, 38.79) and filtered against the *Macaca mulatta* genome (Reference sequence: GCF_000772875) to remove any reads from the host cells before assembly, using Bowtie2. Filtered data was assembled using SPAdes (v3.8.0) [42]. DNA of isolate NL_Conj_Li was also used for long-read sequencing to generate a fully closed genome. To close the genome of NL_Conj_Li, sequencing was repeated with Oxford Nanopore on DNA of the fifth passage. A sequencing library was prepared using Ligation sequencing kit SQK-LSK-109 and sequenced on flowcell type FLO-MIN106D in the MinION Mk1-b, according to the manufacturer’s protocol. Hybrid assembly was performed using Unicycler (v0.4.6) [43]. Annotation of the genomes was generated with Prokka (v1.14.6) [44].

## 5. Conclusions

To conclude, we found a lower *C. caviae* prevalence than had previously been assumed, with 2.7% (7/260) and 8.9% (78/878) in guinea pigs, originating from 6.6% (2/30) and 8.1% (3/37) of husbandries in Switzerland and in The Netherlands, respectively. Genotyping of the complete *ompA* gene of *C. caviae* showed that all Dutch and Swiss sequences were sharing 100% nucleotide identity with the isolated strains from a Dutch patient during a zoonotic transmission from its guinea pig, but different from the *C. caviae* GPIC reference strain. These results might suggest the presence of one circulating strain in the European guinea pig population, that can potentially cause zoonotic transmission.

The majority (88.2% (75/85)) of all *C. caviae*-positive guinea pigs were asymptomatic, which might lead to unnoticed infections and a rapid spread of the pathogen within one husbandry. No *C. caviae* was detected in rabbits, but *C. psittaci* was identified in conjunctival samples from rabbits and rectal samples from guinea pigs. None of the owners displayed severe respiratory signs at the time of sampling. Nevertheless, our results raise concerns about another zoonotic chlamydial strain present in these two animal species and the possibility of novel host species harboring *C. psittaci*. Therefore, caution and general hygiene measures should be applied when handling rodents and lagomorphs, especially if these are exhibiting typical *Chlamydia*-related clinical signs.

## Figures and Tables

**Table 1:** Details on the *Chlamydiaceae*-positive husbandries in the Swiss and Dutch prevalence study.

Swiss Prevalence Study							
Husbandry Number	Type of Husbandry	Number of Animals	Clinical Signs Present (y/n)	Type of Clinical Signs in Guinea Pigs and Rabbits	*Chlamydiaceae-*Positive Swabs (n)/ Total (n)	Range of Ct Values	*C. caviae* Confirmed (n)/ *Chlamydiaceae*-Positive (n)
5	Breeder	22	yes	Subconjunctival fat deposition, serous and mucous ocular discharge, lens opacification, chemosis, crust accumulation	6/76	25.8–35.6	3/6 ^A^
8	Breeder	11	yes	Crust accumulation, serous ocular and nasal discharge	5/23	24–34.1	5/5
18	Private owner	7	yes	Crust accumulation	1/15	34.7	0/1 ^B^
19	Private owner	27	yes	Hyperemia, crust accumulation	1/55	32	0/1 ^B^
21	Private owner	11	yes	Mucous ocular discharge, serous nasal discharge	1/24	36.7	0/1
24	Breeder	18	yes	Mucous and serous nasal discharge, crusts accumulation, corneal opacification	1/37	36.7	0/1 ^B^
26	Private owner	12	yes	Serous nasal discharge, mucous ocular discharge, hyperemia, crusts accumulation	1/26	32.3	0/1 ^B^
28	Private owner	12	yes	Serous ocular discharge, corneal opacification, subconjunctival fat deposition	1/25	34.6	0/1
57	Private owner	1	no	−	1/2	37.6	0/1
**Dutch Prevalence Study**							
**Husbandry Number**	**Type of Husbandry**	**Number of Animals**	**Clinical Signs Present (y/n)**	**Type of Clinical Signs in Guinea Pigs**	***Chlamydiaceae*-Positive Swabs (n)/ Total (n)**	**Range of Ct Values**	***C. caviae* Confirmed (n)/ *Chlamydiaceae*-Positive (n)**
5	Show breeder	28	no	−	1/6	31.1	1/1
13	Show breeder	83	yes	Mucopurulent ocular discharge, pharyngeal stridor, conjunctivitis, corneal edema, corneal lesions, rhonchi, painful mandibular lymph nodes	9/20	24.6–36.3	7/9
19	Show breeder	31	no	−	1/7	37.7	0/1
28	Show breeder	8	no	−	1/2	37.2	0/1
38	Trader	51	yes	Conjunctivitis, mucous and mucopurulent ocular discharge, blepharospasm, mucous nasal discharge, nasal stridor, enlarged mandibular lymph nodes	13/13	22–36.1	11/13

^A^ Insufficient DNA for *C. caviae* typing; ^B^ *C. psittaci* positive samples.

**Table 2:** Details on the *Chlamydiaceae*-, *C. caviae*-, and *C. psittaci-* positive samples of the Swiss and Dutch prevalence study.

	Switzerland					The Netherlands			
	*Chlamydiaceae-*Positive ^A^ (Guinea Pigs/Rabbits)	*C.caviae*-Positive ^B^ (Guinea Pigs/Rabbits)	*C.psittaci*-Positive ^C^ (Guinea Pigs/Rabbits)	*Chlamydiaceae-*Negative (Guinea Pigs/Rabbits)	Total of Collected Samples (Guinea Pigs/Rabbits)	*Chlamydiaceae*-Positive ^A^	*C.caviae*-Positive ^B^	*Chlamydiaceae*-Negative	Total of Collected Samples
Conjunctival samples	11/3	7/0	0/2	282/118	293/121	25	19	175	200
Individual samples	1/1	1/0	0/1	65/10	66/11	0	0	0	0
Pooled samples	10/2	6/0	0/1	217/108	227/110	0	0	0	0
Composite samples	0/0	0/0	0/0	0/0	0/0	25	19	175	200
Rectal samples	4/0	1/0	2/0	256/110	260/110	0	0	0	0
Total	15/3	8/0	2/2	538/228	553/231	25	19	175	200

^A^ Identified by 23S rRNA *Chlamydiaceae*-specific real-time PCR; ^B^ Identified by 16S rRNA PCR, VD4 *C. caviae ompA* PCR and *C. caviae ompA* genotyping, depending on the country; ^C^ Identified by *C. psittaci*-specific qPCR und *C. psittaci ompA* genotyping.

**Table 3:** Properties of the genomic sequences of the two *C. caviae* isolates.

	NL_Conj_Li Chromosome	NL_Conj_Li Plasmid	04DC41 Chromosome	04DC41 Plasmid
**Size**	1,175,666	7532	1,175,594	7659
**Contigs**	1	1	9	1
**GC%**	39.27	33.48	39.26	33.32
**CDS**	987	8	988	8
**rRNA**	3		3	
**tRNA**	38		38	
**ANI to GPIC**	99.55	99.79	99.54	99.84

**Table 4:** Number of animals and origin of samples by country.

	Switzerland						The Netherlands						Total Number of Samples	Total Number of Animals
	Husbandries	Animals	Conjunctival Samples			Rectal Samples	Husbandries	Animals	Conjunctival samples			Rectal Samples		
			Individual	Pooled	Composite				Individual	Pooled	Composite ^1^			
**Guinea Pigs**	**30**	**260**	**66**	**227**	**0**	**260**	**37**	878	0	0	200	0	753	1138
**Rabbits**	34	110	11	110	0	110	0	0	0	0	0	0	231	110
**Total**	64 ^2^	370	414			370	37	878	200			0	984	1248

^1^ Swabs from one conjunctiva with a range of one to six guinea pigs per composite swab; ^2^ Certain husbandries had guinea pigs and rabbits living together in the same enclosure.

**Table 5:** Details on primers, probes and their concentrations for the real-time PCR (qPCR) and conventional PCR (PCR) methods used in this study (eGFP = enhanced green fluorescent protein, IPC = internal positive control, *ompA* = outer membrane protein A).

Method	Target	Primer and Probe	Sequence (5’–3’)	Final Concentration of Primers and Probe in the PCR Mix	Base Pairs for Each Amplicon	Annealing Temperature	References
*Chlamydiaceae* 23S rRNA qPCR ^1^	23S rRNA ^1^	Ch23S-F	CTGAAACCAGTAGCTTATAAGGGGT	500 nM ^A^/1000 nM ^B^	111	60 °C	[34]
		Ch23S-R	ACCTCGCCGTTTAACTTAACTCC				
		Ch23S-p	FAM-CTCATCATGCAAAAGGCACGCCG-TAMRA	200 nM			
	eGFP ^3^	eGFP-1-F	GACCACTACCAGCAGAACAC	200 nM	177		[35]
		eGFP-10-R	CTTGTACAGCTCGTACATGC				
		eGFP-Hex	HEX-AGCACCCAGTCCGCCCTGAGCA-BHQI				
	IPC ^2^	ChMIX3IPC-template (plasmid)	ACCTCGCCGTTTAACTTAACTCCCTGCGCGGATGCTAATGG CACAAGCGCGTCGTTCGTACCTAGAAGGTTTGAAGCACCTT CCCACATAGTGACCGCTTATAAGCTACTGGTTTCAG	200 nM			In-house
		IPC-probe	VIC-CGCGTCGTTCGTACC-MGB-NFQ				
16S rRNA PCR ^3^	16S rRNA	16S IGF	GATGAGGCATGCAAGTCGAACG	300 nM	278	58 °C	[37]
		16S IGR	CCAGTGTTGGCGGTCAATCTCTC				
VD4 *C. caviae ompA* PCR ^1^	VD4 of the *ompA* gene	CCVDF CCVDR	GTCCAGAGCTACATTTGATGC ATTTTGTTGATTTGAAGCGAAGC	500 nM	130	60 °C	[38]
*C. psittaci*-specific qPCR ^3^	*ompA*	CppsOMP1_For	CACTATGTGGGAAGGTGCTTCA	900 nM	76	60 °C	[39]
		CppsOMP1_Rev	CTGCGCGGATGCTAATGG				
		CppsOMP1	FAM-CGCTACTTGGTGTGAC-MGB-NFQ	200 nM			
	eGFP	eGFP_For	GACCACTACCAGCAGAACAC	400 nM	132		[35]
		eGFP_Rev	GAACTCCAGCAGGACCATG				
		eGFP_probe	AGCACCCAGTCCGCCCTGAGCA	200 nM			
*OmpA C. psittaci*-specific PCR ^3^	*ompA*	ompA F (CTU)	ATGAAAAAACTCTTGAAATCGG	200 nM	1050	49 °C	[40]
		ompA rev	TCCTTAGAATCTGAATTGAGC				
*C. caviae* complete *ompA* gene PCR ^1^	*ompA*	ompA_Fw1	GAATAGCGAGCACAAAAAGAAAAGA	500 nM ^A^/400 nM ^B^	1268	59 °C ^A^ 60 °C ^B^	[8]
		ompA_Rv1	GGTTCTGATAGCGGGACAAAAA				
	Additional primers	ompA_Fw3	GCAGAATGGTCCACAAAT#GC	500 nM	498		
		ompA_Rv3	GTTCAATCTATAAGAAAGAGCTAAAC				

^1^ Method and target applied for Swiss and Dutch samples; ^2^ Method and target applied exclusively for the Dutch samples; ^3^ Method and target applied exclusively for the Swiss samples; ^A^Primer concentration and annealing temperature applied for the Swiss methods; ^B^ Primer concentration and annealing temperature applied for the Dutch methods.

## Data Availability

All data supporting reported results are contained in the manuscript and the Supplementary Materials and are available on the server of the Institute of Veterinary Pathology.

## Supplementary Materials

The following are available online at https://www.mdpi.com/article/10.3390/pathogens10101230/s1, Table S1: Details on sample identity, diagnostics performed and subsequent results of each conjunctival and rectal swab sampled in Switzerland (qPCR: real-time PCR; PCR: conventional PCR). Table S2: Details on sample identity, diagnostics performed, and subsequent results of each conjunctival composite swab sampled in The Netherlands (qPCR: real-time PCR; PCR: conventional PCR). Table S3: Sequence comparison of specific genes of interest between strain NL_Conj_Li to strains 04DC41 and GPIC. Table S4: Content of each reaction mix and cycling protocols for the different real-time PCR (qPCR) and conventional PCR (PCR) methods used in this study.
