# Supplementary material for: Chlamydia caviae in Swiss and Dutch Guinea Pigs—Occurrence and Genetic Diversity
Source: Pathogens. 2021 Sep 23;10(10):1230. doi: 10.3390/pathogens10101230 (PMC8539544; doi:10.3390/pathogens10101230)
Supplement: Supplementary file 1 [file pathogens-10-01230-s001.zip › pathogens-1350460-supplementary.pdf]

Supplementary Table 1: Details on sample identity, diagnostics performed and subsequent results of each conjunctival and rectal swab sampled in Switzerland (qPCR: real-time PCR; PCR: conventional PCR).

**Sample number**[A]	**Sample ID**[B]	**Breeder ID**	**Presence of clinical signs (ocular or nasal discharge, ocular pathologies)**	**23S *Chlamydiaceae* qPCR**		**16S PCR and sequencing result (% percentage nucleotide identity)**	**VD4 *C. caviae* PCR**	***C. psittaci* specific qPCR**	***C. psittaci ompA* genotyping**	***C. caviae ompA* genotyping**
				**Result**	**Mean Ct value**					
G1	G1C	1	no	negative	undet	n.d.	n.d.	n.d.	n.d.	n.d.
	G1R	1	no	negative	undet.	n.d.	n.d.	n.d.	n.d.	n.d.
G2	G2C	1	no	negative	undet.	n.d.	n.d.	n.d.	n.d.	n.d.
	G2R	1	no	negative	undet.	n.d.	n.d.	n.d.	n.d.	n.d.
G3	G3C	1	no	negative	undet.	n.d.	n.d.	n.d.	n.d.	n.d.
	G3R	1	no	negative	undet.	n.d.	n.d.	n.d.	n.d.	n.d.
G4	G4C_right	1	yes	negative	undet.	n.d.	n.d.	n.d.	n.d.	n.d.
	G4R	1	yes	negative	undet.	n.d.	n.d.	n.d.	n.d.	n.d.
	G4C_left	1	yes	negative	undet.	n.d.	n.d.	n.d.	n.d.	n.d.
G5	G5C	1	no	negative	undet.	n.d.	n.d.	n.d.	n.d.	n.d.
	G5R	1	no	negative	undet.	n.d.	n.d.	n.d.	n.d.	n.d.
G6	G6C	1	no	negative	undet.	n.d.	n.d.	n.d.	n.d.	n.d.
	G6R	1	no	negative	undet.	n.d.	n.d.	n.d.	n.d.	n.d.
G7	G7C	1	no	negative	undet.	n.d.	n.d.	n.d.	n.d.	n.d.
	G7R	1	no	negative	undet.	n.d.	n.d.	n.d.	n.d.	n.d.
G8	G8C_right	1	yes	negative	undet.	n.d.	n.d.	n.d.	n.d.	n.d.
	G8R	1	yes	negative	undet.	n.d.	n.d.	n.d.	n.d.	n.d.
	G8C_left	1	yes	negative	undet.	n.d.	n.d.	n.d.	n.d.	n.d.
G9	G9C	1	no	negative	undet.	n.d.	n.d.	n.d.	n.d.	n.d.
	G9R	1	no	negative	undet.	n.d.	n.d.	n.d.	n.d.	n.d.
G10	G10C	1	no	negative	undet.	n.d.	n.d.	n.d.	n.d.	n.d.

	G10R	1	no	negative	undet.	n.d.	n.d.	n.d.	n.d.	n.d.
G11	G11C	1	no	negative	undet.	n.d.	n.d.	n.d.	n.d.	n.d.
	G11R	1	no	negative	undet.	n.d.	n.d.	n.d.	n.d.	n.d.
G12	G12C	2	no	negative	undet.	n.d.	n.d.	n.d.	n.d.	n.d.
	G12R	2	no	negative	undet.	n.d.	n.d.	n.d.	n.d.	n.d.
G13	G13C	2	no	negative	undet.	n.d.	n.d.	n.d.	n.d.	n.d.
	G13R	2	no	negative	undet.	n.d.	n.d.	n.d.	n.d.	n.d.
G14	G14C	2	no	negative	undet.	n.d.	n.d.	n.d.	n.d.	n.d.
	G14R	2	no	negative	undet.	n.d.	n.d.	n.d.	n.d.	n.d.
G15	G15C	2	no	negative	undet.	n.d.	n.d.	n.d.	n.d.	n.d.
	G15R	2	no	negative	undet.	n.d.	n.d.	n.d.	n.d.	n.d.
G16	G16C	2	no	negative	undet.	n.d.	n.d.	n.d.	n.d.	n.d.
	G16R	2	no	negative	undet.	n.d.	n.d.	n.d.	n.d.	n.d.
G17	G17C	2	no	negative	undet.	n.d.	n.d.	n.d.	n.d.	n.d.
	G17R	2	no	negative	undet.	n.d.	n.d.	n.d.	n.d.	n.d.
G18	G18C_right	3	yes	negative	undet.	n.d.	n.d.	n.d.	n.d.	n.d.
	G18R	3	yes	negative	undet.	n.d.	n.d.	n.d.	n.d.	n.d.
	G18C_left	3	yes	negative	undet.	n.d.	n.d.	n.d.	n.d.	n.d.
G19	G19C	3	no	negative	undet.	n.d.	n.d.	n.d.	n.d.	n.d.
	G19R	3	no	negative	undet.	n.d.	n.d.	n.d.	n.d.	n.d.
G20	G20C	3	no	negative	undet.	n.d.	n.d.	n.d.	n.d.	n.d.
	G20R	3	no	negative	undet.	n.d.	n.d.	n.d.	n.d.	n.d.
G21	G21C	3	no	negative	undet.	n.d.	n.d.	n.d.	n.d.	n.d.
	G21R	3	no	negative	undet.	n.d.	n.d.	n.d.	n.d.	n.d.
G22	G22C	3	no	negative	undet.	n.d.	n.d.	n.d.	n.d.	n.d.
	G22R	3	no	negative	undet.	n.d.	n.d.	n.d.	n.d.	n.d.
G23	G23C_right	3	no	negative	undet.	n.d.	n.d.	n.d.	n.d.	n.d.
	G23R	3	no	negative	undet.	n.d.	n.d.	n.d.	n.d.	n.d.
	G23C_left	3	no	negative	undet.	n.d.	n.d.	n.d.	n.d.	n.d.
G24	G24C	3	no	negative	undet.	n.d.	n.d.	n.d.	n.d.	n.d.
	G24R	3	no	negative	undet.	n.d.	n.d.	n.d.	n.d.	n.d.

G25	G25C	3	no	negative	undet.	n.d.	n.d.	n.d.	n.d.	n.d.
	G25R	3	no	negative	undet.	n.d.	n.d.	n.d.	n.d.	n.d.
G26	G26C	3	no	negative	undet.	n.d.	n.d.	n.d.	n.d.	n.d.
	G26R	3	no	negative	undet.	n.d.	n.d.	n.d.	n.d.	n.d.
G27	G27C	3	no	negative	undet.	n.d.	n.d.	n.d.	n.d.	n.d.
	G27R	3	no	negative	undet.	n.d.	n.d.	n.d.	n.d.	n.d.
G28	G28C	3	no	negative	undet.	n.d.	n.d.	n.d.	n.d.	n.d.
	G28R	3	no	negative	undet.	n.d.	n.d.	n.d.	n.d.	n.d.
G29	G29C	3	no	negative	undet.	n.d.	n.d.	n.d.	n.d.	n.d.
	G29R	3	no	negative	undet.	n.d.	n.d.	n.d.	n.d.	n.d.
G30	G30C	4	no	negative	undet.	n.d.	n.d.	n.d.	n.d.	n.d.
	G30R	4	no	negative	undet.	n.d.	n.d.	n.d.	n.d.	n.d.
G31	G31C	4	no	negative	undet.	n.d.	n.d.	n.d.	n.d.	n.d.
	G31R	4	no	negative	undet.	n.d.	n.d.	n.d.	n.d.	n.d.
G32	G32C	4	no	negative	undet.	n.d.	n.d.	n.d.	n.d.	n.d.
	G32R	4	no	negative	undet.	n.d.	n.d.	n.d.	n.d.	n.d.
G33	G33C	4	no	negative	undet.	n.d.	n.d.	n.d.	n.d.	n.d.
	G33R	4	no	negative	undet.	n.d.	n.d.	n.d.	n.d.	n.d.
G34	G34C	4	no	negative	undet.	n.d.	n.d.	n.d.	n.d.	n.d.
	G34R	4	no	negative	undet.	n.d.	n.d.	n.d.	n.d.	n.d.
G35	G35C	4	no	negative	undet.	n.d.	n.d.	n.d.	n.d.	n.d.
	G35R	4	no	negative	undet.	n.d.	n.d.	n.d.	n.d.	n.d.
G36	G36C	4	no	negative	undet.	n.d.	n.d.	n.d.	n.d.	n.d.
	G36R	4	no	negative	undet.	n.d.	n.d.	n.d.	n.d.	n.d.
G37	G37C	4	no	negative	undet.	n.d.	n.d.	n.d.	n.d.	n.d.
	G37R	4	no	negative	undet.	n.d.	n.d.	n.d.	n.d.	n.d.
G38	G38C	4	no	negative	undet.	n.d.	n.d.	n.d.	n.d.	n.d.
	G38R	4	no	negative	undet.	n.d.	n.d.	n.d.	n.d.	n.d.
G39	G39C_right	5	yes	negative	undet.	n.d.	n.d.	n.d.	n.d.	n.d.
	G39R	5	yes	negative	undet.	n.d.	n.d.	n.d.	n.d.	n.d.
	G39C_left	5	yes	negative	undet.	n.d.	n.d.	n.d.	n.d.	n.d.

G40	G40C	5	no	negative	undet.	n.d.	n.d.	n.d.	n.d.	n.d.
	G40R	5	no	negative	undet.	n.d.	n.d.	n.d.	n.d.	n.d.
G41	G41C	5	no	negative	undet.	n.d.	n.d.	n.d.	n.d.	n.d.
	G41R	5	no	negative	undet.	n.d.	n.d.	n.d.	n.d.	n.d.
G42	G42C	5	no	negative	undet.	n.d.	n.d.	n.d.	n.d.	n.d.
	G42R	5	no	negative	undet.	n.d.	n.d.	n.d.	n.d.	n.d.
G43	G43C	5	yes	negative	undet.	n.d.	n.d.	n.d.	n.d.	n.d.
	G43R	5	yes	negative	undet.	n.d.	n.d.	n.d.	n.d.	n.d.
G44	G44C	5	no	negative	undet.	n.d.	n.d.	n.d.	n.d.	n.d.
	G44R	5	no	negative	undet.	n.d.	n.d.	n.d.	n.d.	n.d.
G45	G45C	5	no	negative	undet.	n.d.	n.d.	n.d.	n.d.	n.d.
	G45R	5	no	negative	undet.	n.d.	n.d.	n.d.	n.d.	n.d.
G46	G46C	5	no	negative	undet.	n.d.	n.d.	n.d.	n.d.	n.d.
	G46R	5	no	negative	undet.	n.d.	n.d.	n.d.	n.d.	n.d.
G47	G47C_right	5	yes	negative	undet.	n.d.	n.d.	n.d.	n.d.	n.d.
	G47R	5	yes	negative	undet.	n.d.	n.d.	n.d.	n.d.	n.d.
	G47C_left	5	yes	negative	undet.	n.d.	n.d.	n.d.	n.d.	n.d.
G48	G48C	5	yes	negative	undet.	n.d.	n.d.	n.d.	n.d.	n.d.
	G48R	5	yes	negative	undet.	n.d.	n.d.	n.d.	n.d.	n.d.
G49	G49C	5	no	negative	undet.	n.d.	n.d.	n.d.	n.d.	n.d.
	G49R	5	no	negative	undet.	n.d.	n.d.	n.d.	n.d.	n.d.
G50	G50C	5	no	negative	undet.	n.d.	n.d.	n.d.	n.d.	n.d.
	G50R	5	no	negative	undet.	n.d.	n.d.	n.d.	n.d.	n.d.
G51	G51C	5	no	negative	undet.	n.d.	n.d.	n.d.	n.d.	n.d.
	G51R	5	no	negative	undet.	n.d.	n.d.	n.d.	n.d.	n.d.
G52	G52C_right	5	yes	negative	undet.	n.d.	n.d.	n.d.	n.d.	n.d.
	G52R	5	yes	negative	undet.	n.d.	n.d.	n.d.	n.d.	n.d.
	G52C_left	5	yes	negative	undet.	n.d.	n.d.	n.d.	n.d.	n.d.
G53	G53C	5	no	negative	undet.	n.d.	n.d.	n.d.	n.d.	n.d.
	G53R	5	no	negative	undet.	n.d.	n.d.	n.d.	n.d.	n.d.
G54	G54C	5	no	negative	undet.	n.d.	n.d.	n.d.	n.d.	n.d.

	G54R	5	no	negative	undet.	n.d.	n.d.	n.d.	n.d.	n.d.
G55	G55C	5	yes	positive	27.99	99.6% *C. caviae* GPIC 16S (NR_074946.1)	n.d.	n.d.	n.d.	100% *C. caviae* (KY777661)
	G55R	5	yes	positive	31.32	99.62% *C. caviae* GPIC 16S (NR_074946.1)	n.d.	n.d.	n.d.	100% *C. caviae* (KY777661)
G56	G56C_right	5	no	negative	undet.	n.d.	n.d.	n.d.	n.d.	n.d.
	G56R	5	no	negative	undet.	n.d.	n.d.	n.d.	n.d.	n.d.
	G56C_left	5	no	negative	undet.	n.d.	n.d.	n.d.	n.d.	n.d.
G57	G57C	5	no	negative	undet.	n.d.	n.d.	n.d.	n.d.	n.d.
	G57R	5	no	negative	undet.	n.d.	n.d.	n.d.	n.d.	n.d.
G58	G58C_right	5	yes	positive	25.82	99.62% C. *caviae* (NR_074946.1)	n.d.	n.d.	n.d.	100% *C. caviae* (KY777661)
	G58R	5	yes	negative	undet.	n.d.	n.d.	n.d.	n.d.	n.d.
	G58C_left	5	yes	negative	undet.	n.d.	n.d.	n.d.	n.d.	n.d.
G59	G59C_right	5	yes	negative	undet.	n.d.	n.d.	n.d.	n.d.	n.d.
	G59R	5	yes	negative	undet.	n.d.	n.d.	n.d.	n.d.	n.d.
	G59C_left	5	yes	negative	undet.	n.d.	n.d.	n.d.	n.d.	n.d.
G60	G60C_right	5	yes	negative	undet.	n.d.	n.d.	n.d.	n.d.	n.d.
	G60R	5	yes	negative	undet.	n.d.	n.d.	n.d.	n.d.	n.d.
	G60C_left	5	yes	negative	undet.	n.d.	n.d.	n.d.	n.d.	n.d.
G61	G61C_right	5	yes	negative	undet.	n.d.	n.d.	n.d.	n.d.	n.d.
	G61R	5	yes	negative	undet.	n.d.	n.d.	n.d.	n.d.	n.d.
	G61C_left	5	yes	negative	undet.	n.d.	n.d.	n.d.	n.d.	n.d.
G62	G62C	5	no	negative	undet.	n.d.	n.d.	n.d.	n.d.	n.d.
	G62R	5	no	negative	undet.	n.d.	n.d.	n.d.	n.d.	n.d.
G63	G63C	5	no	negative	undet.	n.d.	n.d.	n.d.	n.d.	n.d.
	G63R	5	no	negative	undet.	n.d.	n.d.	n.d.	n.d.	n.d.
G64	G64C	5	no	negative	undet.	n.d.	n.d.	n.d.	n.d.	n.d.
	G64R	5	no	negative	undet.	n.d.	n.d.	n.d.	n.d.	n.d.

G65	G65C	5	no	positive	36.09	n.i.	n.d.[1]	n.d.[1]	n.d.	n.d.
	G65R	5	no	questionable	39.34	n.i.	n.d.	negative	n.d.	n.d.
G66	G66C_right	5	yes	negative	undet.	n.d.	n.d.	n.d.	n.d.	n.d.
	G66R	5	yes	negative	undet.	n.d.	n.d.	n.d.	n.d.	n.d.
	G66C_left	5	yes	negative	undet.	n.d.	n.d.	n.d.	n.d.	n.d.
G67	G67C	5	no	positive	35.58	n.i.	n.d.[1]	n.d.[1]	n.d.	n.d.
	G67R	5	no	negative	undet.	n.d.	n.d.	n.d.	n.d.	n.d.
G68	G68C	5	no	positive	35.64	n.i.	negative	negative	n.d.	n.d.
	G68R	5	no	negative	undet.	n.d.	n.d.	n.d.	n.d.	n.d.
G69	G69C	5	no	negative	undet.	n.d.	n.d.	n.d.	n.d.	n.d.
	G69R	5	no	negative	undet.	n.d.	n.d.	n.d.	n.d.	n.d.
G70	G70C_right	5	yes	negative	undet.	n.d.	n.d.	n.d.	n.d.	n.d.
	G70R	5	yes	negative	undet.	n.d.	n.d.	n.d.	n.d.	n.d.
	G70C_left	5	yes	negative	undet.	n.d.	n.d.	n.d.	n.d.	n.d.
G71	G71C	5	no	negative	undet.	n.d.	n.d.	n.d.	n.d.	n.d.
	G71R	5	no	negative	undet.	n.d.	n.d.	n.d.	n.d.	n.d.
G72	G72C_right	6	yes	negative	undet.	n.d.	n.d.	n.d.	n.d.	n.d.
	G72R	6	yes	negative	undet.	n.d.	n.d.	n.d.	n.d.	n.d.
	G72C_left	6	yes	negative	undet.	n.d.	n.d.	n.d.	n.d.	n.d.
G73	G73C_right	6	yes	negative	undet.	n.d.	n.d.	n.d.	n.d.	n.d.
	G73R	6	yes	negative	undet.	n.d.	n.d.	n.d.	n.d.	n.d.
	G73C_left	6	yes	negative	undet.	n.d.	n.d.	n.d.	n.d.	n.d.
G74	G74C	6	no	negative	undet.	n.d.	n.d.	n.d.	n.d.	n.d.
	G74R	6	no	negative	undet.	n.d.	n.d.	n.d.	n.d.	n.d.
G75	G75C	6	no	negative	undet.	n.d.	n.d.	n.d.	n.d.	n.d.
	G75R	6	no	negative	undet.	n.d.	n.d.	n.d.	n.d.	n.d.
G76	G76C	6	no	negative	undet.	n.d.	n.d.	n.d.	n.d.	n.d.
	G76R	6	no	negative	undet.	n.d.	n.d.	n.d.	n.d.	n.d.
G77	G77C	6	no	negative	undet.	n.d.	n.d.	n.d.	n.d.	n.d.
	G77R	6	no	negative	undet.	n.d.	n.d.	n.d.	n.d.	n.d.
G78	G78C	6	no	negative	undet.	n.d.	n.d.	n.d.	n.d.	n.d.

	G78R	6	no	negative	undet.	n.d.	n.d.	n.d.	n.d.	n.d.
G79	G79C	6	no	negative	undet.	n.d.	n.d.	n.d.	n.d.	n.d.
	G79R	6	no	negative	undet.	n.d.	n.d.	n.d.	n.d.	n.d.
G80	G80C	7	yes	negative	undet.	n.d.	n.d.	n.d.	n.d.	n.d.
	G80R	7	yes	negative	undet.	n.d.	n.d.	n.d.	n.d.	n.d.
G81	G81C	7	yes	negative	undet.	n.d.	n.d.	n.d.	n.d.	n.d.
	G81R	7	yes	negative	undet.	n.d.	n.d.	n.d.	n.d.	n.d.
G82	G82C	7	no	negative	undet.	n.d.	n.d.	n.d.	n.d.	n.d.
	G82R	7	no	negative	undet.	n.d.	n.d.	n.d.	n.d.	n.d.
G83	G83C	7	no	negative	undet.	n.d.	n.d.	n.d.	n.d.	n.d.
	G83R	7	no	negative	undet.	n.d.	n.d.	n.d.	n.d.	n.d.
G84	G84C	7	yes	negative	undet.	n.d.	n.d.	n.d.	n.d.	n.d.
	G84R	7	yes	negative	undet.	n.d.	n.d.	n.d.	n.d.	n.d.
G85	G85C	7	no	negative	undet.	n.d.	n.d.	n.d.	n.d.	n.d.
	G85R	7	no	negative	undet.	n.d.	n.d.	n.d.	n.d.	n.d.
G86	G86C_right	7	yes	negative	undet.	n.d.	n.d.	n.d.	n.d.	n.d.
	G86R	7	yes	negative	undet.	n.d.	n.d.	n.d.	n.d.	n.d.
	G86C_left	7	yes	negative	undet.	n.d.	n.d.	n.d.	n.d.	n.d.
G87	G87C	7	no	negative	undet.	n.d.	n.d.	n.d.	n.d.	n.d.
	G87R	7	no	negative	undet.	n.d.	n.d.	n.d.	n.d.	n.d.
G88	G88C	7	no	negative	undet.	n.d.	n.d.	n.d.	n.d.	n.d.
	G88R	7	no	negative	undet.	n.d.	n.d.	n.d.	n.d.	n.d.
G89	G89C	7	no	negative	undet.	n.d.	n.d.	n.d.	n.d.	n.d.
	G89R	7	no	negative	undet.	n.d.	n.d.	n.d.	n.d.	n.d.
G90	G90C	8	yes	positive	33.78	n.i.	positive	negative	n.d.	100% *C. caviae* (KY777661)
	G90R	8	yes	negative	undet.	n.d.	n.d.	n.d.	n.d.	n.d.
G91	G91C	8	no	negative	undet.	n.d.	n.d.	n.d.	n.d.	n.d.
	G91R	8	no	negative	undet.	n.d.	n.d.	n.d.	n.d.	n.d.
G92	G92C	8	no	positive	33.55	n.i.	positive	negative	n.d.	n.i.
	G92R	8	no	negative	undet.	n.d.	n.d.	n.d.	n.d.	n.d.

G93	G93C	8	no	negative	undet.	n.d.	n.d.	n.d.	n.d.	n.d.
	G93R	8	no	negative	undet.	n.d.	n.d.	n.d.	n.d.	n.d.
G94	G94C	8	no	positive	23.99	99.64% *C. caviae* GPIC 16S (NR_074946.1)	n.d.	n.d.	n.d.	100% *C. caviae* (KY777661)
	G94R	8	no	negative	undet.	n.d.	n.d.	n.d.	n.d.	n.d.
G95	G95C	8	yes	positive	32.19	99.61% *C. caviae* GPIC 16S (NR_074946.1)	n.d.	n.d.	n.d.	n.i..
	G95R	8	yes	negative	undet.	n.d.	n.d.	n.d.	n.d.	n.d.
G96	G96C	8	no	positive	34.14	n.i.	positive	negative	n.d.	n.i.
	G96R	8	no	negative	undet.	n.d.	n.d.	n.d.	n.d.	n.d.
G97	G97C	8	yes	negative	undet.	n.d.	n.d.	n.d.	n.d.	n.d.
	G97R	8	yes	negative	undet	n.d.	n.d.	n.d.	n.d.	n.d.
G98	G98C_right	8	yes	negative	undet	n.d.	n.d.	n.d.	n.d.	n.d.
	G98R	8	yes	negative	undet	n.d.	n.d.	n.d.	n.d.	n.d.
	G98C_left	8	yes	negative	undet	n.d.	n.d.	n.d.	n.d.	n.d.
G99	G99C	8	no	negative	undet	n.d.	n.d.	n.d.	n.d.	n.d.
	G99R	8	no	negative	undet	n.d.	n.d.	n.d.	n.d.	n.d.
G100	G100C	8	no	negative	undet	n.d.	n.d.	n.d.	n.d.	n.d.
	G100R	8	no	negative	undet	n.d.	n.d.	n.d.	n.d.	n.d.
G101	G101C	9	no	negative	undet.	n.d.	n.d.	n.d.	n.d.	n.d.
	G101R	9	no	negative	undet.	n.d.	n.d.	n.d.	n.d.	n.d.
G102	G102C	9	no	negative	undet.	n.d.	n.d.	n.d.	n.d.	n.d.
	G102R	9	no	negative	undet.	n.d.	n.d.	n.d.	n.d.	n.d.
G103	G103C	9	no	negative	undet.	n.d.	n.d.	n.d.	n.d.	n.d.
	G103R	9	no	negative	undet.	n.d.	n.d.	n.d.	n.d.	n.d.
G104	G104C	9	no	negative	undet.	n.d.	n.d.	n.d.	n.d.	n.d.
	G104R	9	no	negative	undet.	n.d.	n.d.	n.d.	n.d.	n.d.
G105	G105C_right	9	yes	negative	undet.	n.d.	n.d.	n.d.	n.d.	n.d.
	G105R	9	yes	negative	undet.	n.d.	n.d.	n.d.	n.d.	n.d.

	G105C_left	9	yes	negative	undet.	n.d.	n.d.	n.d.	n.d.	n.d.	
G106	G106C	9	no	negative	undet.	n.d.	n.d.	n.d.	n.d.	n.d.	
	G106R	9	no	negative	undet.	n.d.	n.d.	n.d.	n.d.	n.d.	
G107	G107C	9	no	negative	undet.	n.d.	n.d.	n.d.	n.d.	n.d.	
	G107R	9	no	negative	undet.	n.d.	n.d.	n.d.	n.d.	n.d.	
G108	G108C	9	no	negative	undet.	n.d.	n.d.	n.d.	n.d.	n.d.	
	G108R	9	no	negative	undet.	n.d.	n.d.	n.d.	n.d.	n.d.	
G109	G109C	9	no	negative	undet.	n.d.	n.d.	n.d.	n.d.	n.d.	
	G109R	9	no	negative	undet.	n.d.	n.d.	n.d.	n.d.	n.d.	
G100	G110C	9	no	negative	undet.	n.d.	n.d.	n.d.	n.d.	n.d.	
	G110R	9	no	negative	undet.	n.d.	n.d.	n.d.	n.d.	n.d.	
G111	G111C	9	no	negative	undet.	n.d.	n.d.	n.d.	n.d.	n.d.	
	G111R	9	no	negative	undet.	n.d.	n.d.	n.d.	n.d.	n.d.	
G112	G112C	9	no	negative	undet.	n.d.	n.d.	n.d.	n.d.	n.d.	
	G112R	9	no	negative	undet.	n.d.	n.d.	n.d.	n.d.	n.d.	
G113	G113C_right	10	yes	negative	undet.	n.d.	n.d.	n.d.	n.d.	n.d.	
	G113R	10	yes	negative	undet.	n.d.	n.d.	n.d.	n.d.	n.d.	
	G113C_left	10	yes	negative	undet.	n.d.	n.d.	n.d.	n.d.	n.d.	
G114	G114C	10	no	negative	undet.	n.d.	n.d.	n.d.	n.d.	n.d.	
	G114R	10	no	negative	undet.	n.d.	n.d.	n.d.	n.d.	n.d.	
G115	G115C_right	11	yes	negative	undet.	n.d.	n.d.	n.d.	n.d.	n.d.	
	G115R	11	yes	negative	undet.	n.d.	n.d.	n.d.	n.d.	n.d.	
	G115C_left	11	yes	negative	undet.	n.d.	n.d.	n.d.	n.d.	n.d.	
G116	G116C_right	12	yes	negative	undet.	n.d.	n.d.	n.d.	n.d.	n.d.	
	G116R	12	yes	negative	undet.	n.d.	n.d.	n.d.	n.d.	n.d.	
	G116C_left	12	yes	negative	undet.	n.d.	n.d.	n.d.	n.d.	n.d.	
G117	G117C	12	no	negative	undet.	n.d.	n.d.	n.d.	n.d.	n.d.	
	G117R	12	no	negative	undet.	n.d.	n.d.	n.d.	n.d.	n.d.	
G118	G118C_right	13	yes	negative	undet.	n.d.	n.d.	n.d.	n.d.	n.d.	
	G118R	13	yes	negative	undet.	n.d.	n.d.	n.d.	n.d.	n.d.	
	G118C_left	13	yes	negative	undet.	n.d.	n.d.	n.d.	n.d.	n.d.	

G119	G119C	13	no	negative	undet.	n.d.	n.d.	n.d.	n.d.	n.d.
	G119R	13	no	negative	undet.	n.d.	n.d.	n.d.	n.d.	n.d.
G120	G120C	14	no	negative	undet.	n.d.	n.d.	n.d.	n.d.	n.d.
	G120R	14	no	negative	undet.	n.d.	n.d.	n.d.	n.d.	n.d.
G121	G121C	14	no	negative	undet.	n.d.	n.d.	n.d.	n.d.	n.d.
	G121R	14	no	negative	undet.	n.d.	n.d.	n.d.	n.d.	n.d.
G122	G122C	14	yes	negative	undet.	n.d.	n.d.	n.d.	n.d.	n.d.
	G122R	14	yes	negative	undet.	n.d.	n.d.	n.d.	n.d.	n.d.
G123	G123C	14	no	negative	undet.	n.d.	n.d.	n.d.	n.d.	n.d.
	G123R	14	no	negative	undet.	n.d.	n.d.	n.d.	n.d.	n.d.
G124	G124C	14	no	negative	undet.	n.d.	n.d.	n.d.	n.d.	n.d.
	G124R	14	no	negative	undet.	n.d.	n.d.	n.d.	n.d.	n.d.
G125	G125C	14	yes	negative	undet.	n.d.	n.d.	n.d.	n.d.	n.d.
	G125R	14	yes	negative	undet.	n.d.	n.d.	n.d.	n.d.	n.d.
G126	G126C	14	yes	negative	undet.	n.d.	n.d.	n.d.	n.d.	n.d.
	G126R	14	yes	negative	undet.	n.d.	n.d.	n.d.	n.d.	n.d.
G127	G127C	14	no	negative	undet.	n.d.	n.d.	n.d.	n.d.	n.d.
	G127R	14	no	negative	undet.	n.d.	n.d.	n.d.	n.d.	n.d.
G128	G128C_right	14	yes	negative	undet.	n.d.	n.d.	n.d.	n.d.	n.d.
	G128R	14	yes	negative	undet.	n.d.	n.d.	n.d.	n.d.	n.d.
	G128C_left	14	yes	negative	undet.	n.d.	n.d.	n.d.	n.d.	n.d.
G129	G129C	14	no	negative	undet.	n.d.	n.d.	n.d.	n.d.	n.d.
	G129R	14	no	negative	undet.	n.d.	n.d.	n.d.	n.d.	n.d.
G130	G130C	14	yes	negative	undet.	n.d.	n.d.	n.d.	n.d.	n.d.
	G130R	14	yes	negative	undet.	n.d.	n.d.	n.d.	n.d.	n.d.
G131	G131C	14	no	negative	undet.	n.d.	n.d.	n.d.	n.d.	n.d.
	G131R	14	no	negative	undet.	n.d.	n.d.	n.d.	n.d.	n.d.
G132	G132C	14	no	negative	undet.	n.d.	n.d.	n.d.	n.d.	n.d.
	G132R	14	no	negative	undet.	n.d.	n.d.	n.d.	n.d.	n.d.
G133	G133C	14	no	negative	undet.	n.d.	n.d.	n.d.	n.d.	n.d.
	G133R	14	no	negative	undet.	n.d.	n.d.	n.d.	n.d.	n.d.

G134	G134C_right	14	yes	negative	undet.	n.d.	n.d.	n.d.	n.d.	n.d.
	G134R	14	yes	negative	undet.	n.d.	n.d.	n.d.	n.d.	n.d.
	G134C_left	14	yes	negative	undet.	n.d.	n.d.	n.d.	n.d.	n.d.
G135	G135C	14	no	negative	undet.	n.d.	n.d.	n.d.	n.d.	n.d.
	G135R	14	no	negative	undet.	n.d.	n.d.	n.d.	n.d.	n.d.
G136	G136C	15	no	negative	38.47	n.d.	n.d.	n.d.	n.d.	n.d.
	G136R	15	no	negative	undet.	n.d.	n.d.	n.d.	n.d.	n.d.
G137	G137C	15	no	negative	undet.	n.d.	n.d.	n.d.	n.d.	n.d.
	G137R	15	no	negative	undet.	n.d.	n.d.	n.d.	n.d.	n.d.
G138	G138C	15	yes	negative	undet.	n.d.	n.d.	n.d.	n.d.	n.d.
	G138R	15	yes	negative	undet.	n.d.	n.d.	n.d.	n.d.	n.d.
G139	G139C_right	15	yes	negative	undet.	n.d.	n.d.	n.d.	n.d.	n.d.
	G139R	15	yes	negative	undet.	n.d.	n.d.	n.d.	n.d.	n.d.
	G139C_left	15	yes	negative	undet.	n.d.	n.d.	n.d.	n.d.	n.d.
G140	G140C	15	no	negative	undet.	n.d.	n.d.	n.d.	n.d.	n.d.
	G140R	15	no	negative	undet.	n.d.	n.d.	n.d.	n.d.	n.d.
G141	G141C_right	15	yes	negative	undet.	n.d.	n.d.	n.d.	n.d.	n.d.
	G141R	15	yes	negative	undet.	n.d.	n.d.	n.d.	n.d.	n.d.
	G141C_left	15	yes	negative	undet.	n.d.	n.d.	n.d.	n.d.	n.d.
G142	G142C	15	no	negative	undet.	n.d.	n.d.	n.d.	n.d.	n.d.
	G142R	15	no	negative	undet.	n.d.	n.d.	n.d.	n.d.	n.d.
G143	G143C_right	15	yes	negative	undet.	n.d.	n.d.	n.d.	n.d.	n.d.
	G143R	15	yes	negative	undet.	n.d.	n.d.	n.d.	n.d.	n.d.
	G143C_left	15	yes	negative	undet.	n.d.	n.d.	n.d.	n.d.	n.d.
G144	G144C	15	yes	negative	undet.	n.d.	n.d.	n.d.	n.d.	n.d.
	G144R	15	yes	negative	undet.	n.d.	n.d.	n.d.	n.d.	n.d.
G145	G145C	15	yes	negative	undet.	n.d.	n.d.	n.d.	n.d.	n.d.
	G145R	15	yes	negative	undet.	n.d.	n.d.	n.d.	n.d.	n.d.
G146	G146C_right	15	yes	negative	undet.	n.d.	n.d.	n.d.	n.d.	n.d.
	G146R	15	yes	negative	undet.	n.d.	n.d.	n.d.	n.d.	n.d.
	G146C_left	15	yes	negative	undet.	n.d.	n.d.	n.d.	n.d.	n.d.

G147	G147C	15	yes	negative	undet.	n.d.	n.d.	n.d.	n.d.	n.d.
	G147R	15	yes	negative	undet.	n.d.	n.d.	n.d.	n.d.	n.d.
G148	G148C	16	yes	negative	undet.	n.d.	n.d.	n.d.	n.d.	n.d.
	G148R	16	yes	negative	undet.	n.d.	n.d.	n.d.	n.d.	n.d.
G149	G149C	16	no	negative	undet.	n.d.	n.d.	n.d.	n.d.	n.d.
	G149R	16	no	negative	undet.	n.d.	n.d.	n.d.	n.d.	n.d.
G150	G150C	16	no	negative	undet.	n.d.	n.d.	n.d.	n.d.	n.d.
	G150R	16	no	negative	undet.	n.d.	n.d.	n.d.	n.d.	n.d.
G151	G151C	17	no	negative	undet.	n.d.	n.d.	n.d.	n.d.	n.d.
	G151R	17	no	negative	undet.	n.d.	n.d.	n.d.	n.d.	n.d.
G152	G152C	17	no	negative	undet.	n.d.	n.d.	n.d.	n.d.	n.d.
	G152R	17	no	negative	undet.	n.d.	n.d.	n.d.	n.d.	n.d.
G153	G153C	17	yes	negative	undet.	n.d.	n.d.	n.d.	n.d.	n.d.
	G153R	17	yes	negative	undet.	n.d.	n.d.	n.d.	n.d.	n.d.
G154	G154C	17	no	negative	undet.	n.d.	n.d.	n.d.	n.d.	n.d.
	G154R	17	no	negative	undet.	n.d.	n.d.	n.d.	n.d.	n.d.
G155	G155C	17	no	negative	undet.	n.d.	n.d.	n.d.	n.d.	n.d.
	G155R	17	no	negative	undet.	n.d.	n.d.	n.d.	n.d.	n.d.
G156	G156C	17	no	negative	undet.	n.d.	n.d.	n.d.	n.d.	n.d.
	G156R	17	no	negative	undet.	n.d.	n.d.	n.d.	n.d.	n.d.
G157	G157C	17	no	negative	39.63	n.d.	n.d.	n.d.	n.d.	n.d.
	G157R	17	no	negative	undet.	n.d.	n.d.	n.d.	n.d.	n.d.
G158	G158C	18	no	negative	undet.	n.d.	n.d.	n.d.	n.d.	n.d.
	G158R	18	no	negative	undet.	n.d.	n.d.	n.d.	n.d.	n.d.
G159	G159C	18	no	negative	undet.	n.d.	n.d.	n.d.	n.d.	n.d.
	G159R	18	no	negative	undet.	n.d.	n.d.	n.d.	n.d.	n.d.
G160	G160C	18	no	negative	undet.	n.d.	n.d.	n.d.	n.d.	n.d.
	G160R	18	no	negative	undet.	n.d.	n.d.	n.d.	n.d.	n.d.
G161	G161C	18	no	negative	undet.	n.d.	n.d.	n.d.	n.d.	n.d.

	G161R	18	no	positive	34.68	99.63% *C. psittaci* (CP003790.1)	n.d.	positive	99.45% *C. psittaci* 84/55 (CP003790.1)	n.d.
G162	G162C	18	no	negative	undet.	n.d.	n.d.	n.d.	n.d.	n.d.
	G162R	18	no	negative	undet.	n.d.	n.d.	n.d.	n.d.	n.d.
G163	G163C_right	18	yes	negative	undet.	n.d.	n.d.	n.d.	n.d.	n.d.
	G163R	18	yes	negative	undet.	n.d.	n.d.	n.d.	n.d.	n.d.
	G163C_left	18	yes	negative	undet.	n.d.	n.d.	n.d.	n.d.	n.d.
G164	G164C	19	no	negative	undet.	n.d.	n.d.	n.d.	n.d.	n.d.
	G164R	19	no	negative	undet.	n.d.	n.d.	n.d.	n.d.	n.d.
G165	G165C	19	no	negative	undet.	n.d.	n.d.	n.d.	n.d.	n.d.
	G165R	19	no	negative	undet.	n.d.	n.d.	n.d.	n.d.	n.d.
G166	G166C	19	no	negative	undet.	n.d.	n.d.	n.d.	n.d.	n.d.
	G166R	19	no	negative	undet.	n.d.	n.d.	n.d.	n.d.	n.d.
G167	G167C	19	no	negative	undet.	n.d.	n.d.	n.d.	n.d.	n.d.
	G167R	19	no	negative	undet.	n.d.	n.d.	n.d.	n.d.	n.d.
G168	G168C	19	no	negative	undet.	n.d.	n.d.	n.d.	n.d.	n.d.
	G168R	19	no	negative	undet.	n.d.	n.d.	n.d.	n.d.	n.d.
G169	G169C	19	no	negative	undet.	n.d.	n.d.	n.d.	n.d.	n.d.
	G169R	19	no	negative	undet.	n.d.	n.d.	n.d.	n.d.	n.d.
G170	G170C	19	no	negative	undet.	n.d.	n.d.	n.d.	n.d.	n.d.
	G170R	19	no	negative	undet.	n.d.	n.d.	n.d.	n.d.	n.d.
G171	G171C	19	no	negative	undet.	n.d.	n.d.	n.d.	n.d.	n.d.
	G171R	19	no	negative	undet.	n.d.	n.d.	n.d.	n.d.	n.d.
G172	G172C	19	no	negative	undet.	n.d.	n.d.	n.d.	n.d.	n.d.
	G172R	19	no	negative	undet.	n.d.	n.d.	n.d.	n.d.	n.d.
G173	G173C	19	no	negative	undet.	n.d.	n.d.	n.d.	n.d.	n.d.
	G173R	19	no	negative	undet.	n.d.	n.d.	n.d.	n.d.	n.d.
G174	G174C	19	no	negative	undet.	n.d.	n.d.	n.d.	n.d.	n.d.
	G174R	19	no	negative	undet.	n.d.	n.d.	n.d.	n.d.	n.d.
G175	G175C	19	no	negative	undet.	n.d.	n.d.	n.d.	n.d.	n.d.

	G175R	19	no	negative	undet.	n.d.	n.d.	n.d.	n.d.	n.d.
G176	G176C	19	no	negative	undet.	n.d.	n.d.	n.d.	n.d.	n.d.
	G176R	19	no	negative	undet.	n.d.	n.d.	n.d.	n.d.	n.d.
G177	G177C_right	19	yes	negative	undet.	n.d.	n.d.	n.d.	n.d.	n.d.
	G177R	19	yes	negative	undet.	n.d.	n.d.	n.d.	n.d.	n.d.
	G177C_left	19	yes	negative	undet.	n.d.	n.d.	n.d.	n.d.	n.d.
G178	G178C	19	no	negative	undet.	n.d.	n.d.	n.d.	n.d.	n.d.
	G178R	19	no	negative	undet.	n.d.	n.d.	n.d.	n.d.	n.d.
G179	G179C	19	no	negative	undet.	n.d.	n.d.	n.d.	n.d.	n.d.
	G179R	19	no	negative	undet.	n.d.	n.d.	n.d.	n.d.	n.d.
G180	G180C	19	yes	negative	undet.	n.d.	n.d.	n.d.	n.d.	n.d.
	G180R	19	yes	negative	undet.	n.d.	n.d.	n.d.	n.d.	n.d.
G181	G181C	19	yes	negative	undet.	n.d.	n.d.	n.d.	n.d.	n.d.
	G181R	19	yes	negative	undet.	n.d.	n.d.	n.d.	n.d.	n.d.
G182	G182C	19	no	negative	undet.	n.d.	n.d.	n.d.	n.d.	n.d.
	G182R	19	no	negative	undet.	n.d.	n.d.	n.d.	n.d.	n.d.
G183	G183C	19	no	negative	undet.	n.d.	n.d.	n.d.	n.d.	n.d.
	G183R	19	no	negative	undet.	n.d.	n.d.	n.d.	n.d.	n.d.
G184	G184C	19	no	negative	undet.	n.d.	n.d.	n.d.	n.d.	n.d.
	G184R	19	no	positive	32.04	n.i.	n.d.	positive	99.63% *C. psittaci* 84/55 (CP003790.1)	n.d.
G185	G185C	19	no	negative	undet.	n.d.	n.d.	n.d.	n.d.	n.d.
	G185R	19	no	negative	undet.	n.d.	n.d.	n.d.	n.d.	n.d.
G186	G186C	20	no	negative	undet.	n.d.	n.d.	n.d.	n.d.	n.d.
	G186R	20	no	negative	undet.	n.d.	n.d.	n.d.	n.d.	n.d.
G187	G187C	20	yes	negative	undet.	n.d.	n.d.	n.d.	n.d.	n.d.
	G187R	20	yes	negative	undet.	n.d.	n.d.	n.d.	n.d.	n.d.
G188	G188C	20	yes	negative	undet.	n.d.	n.d.	n.d.	n.d.	n.d.
	G188R	20	yes	negative	undet.	n.d.	n.d.	n.d.	n.d.	n.d.
G189	G189C	20	no	negative	undet.	n.d.	n.d.	n.d.	n.d.	n.d.

	G189R	20	no	negative	undet.	n.d.	n.d.	n.d.	n.d.	n.d.
G190	G190C	20	no	negative	undet.	n.d.	n.d.	n.d.	n.d.	n.d.
	G190R	20	no	negative	undet.	n.d.	n.d.	n.d.	n.d.	n.d.
G191	G191C	21	no	negative	undet.	n.d.	n.d.	n.d.	n.d.	n.d.
	G191R	21	no	positive	36.71	n.i.	negative	negative	n.d.	n.d.
G192	G192C	21	no	negative	undet.	n.d.	n.d.	n.d.	n.d.	n.d.
	G192R	21	no	negative	undet.	n.d.	n.d.	n.d.	n.d.	n.d.
G193	G193C	21	no	negative	undet.	n.d.	n.d.	n.d.	n.d.	n.d.
	G193R	21	no	negative	undet.	n.d.	n.d.	n.d.	n.d.	n.d.
G194	G194C	21	no	negative	undet.	n.d.	n.d.	n.d.	n.d.	n.d.
	G194R	21	no	negative	undet.	n.d.	n.d.	n.d.	n.d.	n.d.
G195	G195C	21	no	negative	undet.	n.d.	n.d.	n.d.	n.d.	n.d.
	G195R	21	no	negative	undet.	n.d.	n.d.	n.d.	n.d.	n.d.
G196	G196C	21	yes	negative	undet.	n.d.	n.d.	n.d.	n.d.	n.d.
	G196R	21	yes	negative	undet.	n.d.	n.d.	n.d.	n.d.	n.d.
G197	G197C	22	no	negative	undet.	n.d.	n.d.	n.d.	n.d.	n.d.
	G197R	22	no	negative	undet.	n.d.	n.d.	n.d.	n.d.	n.d.
G198	G198C	22	yes	negative	undet.	n.d.	n.d.	n.d.	n.d.	n.d.
	G198R	22	yes	negative	undet.	n.d.	n.d.	n.d.	n.d.	n.d.
G199	G199C	24	no	negative	undet.	n.d.	n.d.	n.d.	n.d.	n.d.
	G199R	24	no	negative	undet.	n.d.	n.d.	n.d.	n.d.	n.d.
G200	G200C	24	no	negative	undet.	n.d.	n.d.	n.d.	n.d.	n.d.
	G200R	24	no	negative	undet.	n.d.	n.d.	n.d.	n.d.	n.d.
G201	G201C	24	no	negative	undet.	n.d.	n.d.	n.d.	n.d.	n.d.
	G201R	24	no	negative	undet.	n.d.	n.d.	n.d.	n.d.	n.d.
G202	G202C	24	yes	negative	undet.	n.d.	n.d.	n.d.	n.d.	n.d.
	G202R	24	yes	negative	undet.	n.d.	n.d.	n.d.	n.d.	n.d.
G203	G203C	24	no	negative	undet.	n.d.	n.d.	n.d.	n.d.	n.d.
	G203R	24	no	negative	undet.	n.d.	n.d.	n.d.	n.d.	n.d.
G204	G204C	24	no	negative	undet.	n.d.	n.d.	n.d.	n.d.	n.d.
	G204R	24	no	negative	undet.	n.d.	n.d.	n.d.	n.d.	n.d.

G205	G205C	24	no	negative	undet.	n.d.	n.d.	n.d.	n.d.	n.d.
	G205R	24	no	negative	undet.	n.d.	n.d.	n.d.	n.d.	n.d.
G206	G206C	24	no	negative	undet.	n.d.	n.d.	n.d.	n.d.	n.d.
	G206R	24	no	negative	undet.	n.d.	n.d.	n.d.	n.d.	n.d.
G207	G207C	24	no	negative	undet.	n.d.	n.d.	n.d.	n.d.	n.d.
	G207R	24	no	negative	undet.	n.d.	n.d.	n.d.	n.d.	n.d.
G208	G208C	24	no	negative	undet.	n.d.	n.d.	n.d.	n.d.	n.d.
	G208R	24	no	negative	undet.	n.d.	n.d.	n.d.	n.d.	n.d.
G209	G209C	24	no	negative	undet.	n.d.	n.d.	n.d.	n.d.	n.d.
	G209R	24	no	negative	undet.	n.d.	n.d.	n.d.	n.d.	n.d.
G210	G210C	24	no	negative	undet.	n.d.	n.d.	n.d.	n.d.	n.d.
	G210R	24	no	negative	undet.	n.d.	n.d.	n.d.	n.d.	n.d.
G211	G211C	25	no	negative	undet.	n.d.	n.d.	n.d.	n.d.	n.d.
	G211R	25	no	negative	undet.	n.d.	n.d.	n.d.	n.d.	n.d.
G212	G212C_right	25	no	negative	undet.	n.d.	n.d.	n.d.	n.d.	n.d.
	G212R	25	no	negative	undet.	n.d.	n.d.	n.d.	n.d.	n.d.
	G212C_left	25	no	negative	undet.	n.d.	n.d.	n.d.	n.d.	n.d.
G213	G213C	25	no	negative	undet.	n.d.	n.d.	n.d.	n.d.	n.d.
	G213R	25	no	negative	undet.	n.d.	n.d.	n.d.	n.d.	n.d.
G214	G214C	25	no	negative	undet.	n.d.	n.d.	n.d.	n.d.	n.d.
	G214R	25	no	negative	undet.	n.d.	n.d.	n.d.	n.d.	n.d.
G215	G215C	25	no	negative	undet.	n.d.	n.d.	n.d.	n.d.	n.d.
	G215R	25	no	negative	undet.	n.d.	n.d.	n.d.	n.d.	n.d.
G216	G216C	25	no	negative	undet.	n.d.	n.d.	n.d.	n.d.	n.d.
	G216R	25	no	negative	undet.	n.d.	n.d.	n.d.	n.d.	n.d.
G217	G217C	25	no	negative	undet.	n.d.	n.d.	n.d.	n.d.	n.d.
	G217R	25	no	negative	undet.	n.d.	n.d.	n.d.	n.d.	n.d.
G218	G218C	25	yes	negative	undet.	n.d.	n.d.	n.d.	n.d.	n.d.
	G218R	25	yes	negative	undet.	n.d.	n.d.	n.d.	n.d.	n.d.
G219	G219C	25	no	negative	undet.	n.d.	n.d.	n.d.	n.d.	n.d.
	G219R	25	no	negative	undet.	n.d.	n.d.	n.d.	n.d.	n.d.

G220	G220C	25	yes	negative	undet.	n.d.	n.d.	n.d.	n.d.	n.d.
	G220R	25	yes	negative	undet.	n.d.	n.d.	n.d.	n.d.	n.d.
G221	G221C	26	yes	negative	undet.	n.d.	n.d.	n.d.	n.d.	n.d.
	G221R	26	yes	negative	undet.	n.d.	n.d.	n.d.	n.d.	n.d.
G222	G222C	26	no	negative	undet.	n.d.	n.d.	n.d.	n.d.	n.d.
	G222R	26	no	negative	undet.	n.d.	n.d.	n.d.	n.d.	n.d.
G223	G223C	26	no	negative	undet.	n.d.	n.d.	n.d.	n.d.	n.d.
	G223R	26	no	negative	undet.	n.d.	n.d.	n.d.	n.d.	n.d.
G224	G224C	27	no	negative	undet.	n.d.	n.d.	n.d.	n.d.	n.d.
	G224R	27	no	negative	undet.	n.d.	n.d.	n.d.	n.d.	n.d.
G225	G225C	27	yes	negative	undet.	n.d.	n.d.	n.d.	n.d.	n.d.
	G225R	27	yes	negative	undet.	n.d.	n.d.	n.d.	n.d.	n.d.
G226	G226C	27	yes	negative	undet.	n.d.	n.d.	n.d.	n.d.	n.d.
	G226R	27	yes	negative	undet.	n.d.	n.d.	n.d.	n.d.	n.d.
G227	G227C	27	no	negative	undet.	n.d.	n.d.	n.d.	n.d.	n.d.
	G227R	27	no	negative	undet.	n.d.	n.d.	n.d.	n.d.	n.d.
G228	G228C	27	yes	negative	undet.	n.d.	n.d.	n.d.	n.d.	n.d.
	G228R	27	yes	negative	undet.	n.d.	n.d.	n.d.	n.d.	n.d.
G229	G229C_right	28	yes	negative	undet.	n.d.	n.d.	n.d.	n.d.	n.d.
	G229R	28	yes	negative	undet.	n.d.	n.d.	n.d.	n.d.	n.d.
	G229C_left	28	yes	negative	undet.	n.d.	n.d.	n.d.	n.d.	n.d.
G230	G230C	28	yes	negative	undet.	n.d.	n.d.	n.d.	n.d.	n.d.
	G230R	28	yes	negative	undet.	n.d.	n.d.	n.d.	n.d.	n.d.
G231	G231C	28	no	negative	undet.	n.d.	n.d.	n.d.	n.d.	n.d.
	G231R	28	no	negative	undet.	n.d.	n.d.	n.d.	n.d.	n.d.
G232	G232C	28	no	negative	undet.	n.d.	n.d.	n.d.	n.d.	n.d.
	G232R	28	no	negative	undet.	n.d.	n.d.	n.d.	n.d.	n.d.
G233	G233C	28	yes	negative	undet.	n.d.	n.d.	n.d.	n.d.	n.d.
	G233R	28	yes	negative	undet.	n.d.	n.d.	n.d.	n.d.	n.d.
G234	G234C	28	no	negative	undet.	n.d.	n.d.	n.d.	n.d.	n.d.
	G234R	28	no	negative	undet.	n.d.	n.d.	n.d.	n.d.	n.d.

G235	G235C	28	no	negative	undet.	n.d.	n.d.	n.d.	n.d.	n.d.
	G235R	28	no	negative	undet.	n.d.	n.d.	n.d.	n.d.	n.d.
G236	G236C	28	no	negative	undet.	n.d.	n.d.	n.d.	n.d.	n.d.
	G236R	28	no	negative	undet.	n.d.	n.d.	n.d.	n.d.	n.d.
G237	G237C	28	yes	negative	undet.	n.d.	n.d.	n.d.	n.d.	n.d.
	G237R	28	yes	negative	undet.	n.d.	n.d.	n.d.	n.d.	n.d.
G238	G238C	28	no	positive	34.63	n.i.	negative	negative	n.d.	n.d.
	G238R	28	no	negative	undet.	n.d.	n.d.	n.d.	n.d.	n.d.
G239	G239C	28	no	negative	undet.	n.d.	n.d.	n.d.	n.d.	n.d.
	G239R	28	no	negative	undet.	n.d.	n.d.	n.d.	n.d.	n.d.
G240	G240C	28	no	negative	undet.	n.d.	n.d.	n.d.	n.d.	n.d.
	G240R	28	no	negative	undet.	n.d.	n.d.	n.d.	n.d.	n.d.
G241	G241C	29	no	negative	undet.	n.d.	n.d.	n.d.	n.d.	n.d.
	G241R	29	no	negative	undet.	n.d.	n.d.	n.d.	n.d.	n.d.
G242	G242C	29	no	negative	undet.	n.d.	n.d.	n.d.	n.d.	n.d.
	G242R	29	no	negative	undet.	n.d.	n.d.	n.d.	n.d.	n.d.
G243	G243C	29	no	negative	undet.	n.d.	n.d.	n.d.	n.d.	n.d.
	G243R	29	no	negative	undet.	n.d.	n.d.	n.d.	n.d.	n.d.
G244	G244C	29	no	negative	undet.	n.d.	n.d.	n.d.	n.d.	n.d.
	G244R	29	no	negative	undet.	n.d.	n.d.	n.d.	n.d.	n.d.
G245	G245C	29	yes	negative	undet.	n.d.	n.d.	n.d.	n.d.	n.d.
	G245R	29	yes	negative	undet.	n.d.	n.d.	n.d.	n.d.	n.d.
G246	G246C	29	no	negative	undet.	n.d.	n.d.	n.d.	n.d.	n.d.
	G246R	29	no	negative	undet.	n.d.	n.d.	n.d.	n.d.	n.d.
G247	G247C	29	no	negative	undet.	n.d.	n.d.	n.d.	n.d.	n.d.
	G247R	29	no	negative	undet.	n.d.	n.d.	n.d.	n.d.	n.d.
G248	G248C	29	no	negative	undet.	n.d.	n.d.	n.d.	n.d.	n.d.
	G248R	29	no	negative	undet.	n.d.	n.d.	n.d.	n.d.	n.d.
G249	G249C	30	no	negative	undet.	n.d.	n.d.	n.d.	n.d.	n.d.
	G249R	30	no	negative	undet.	n.d.	n.d.	n.d.	n.d.	n.d.
G250	G250C	30	no	negative	undet.	n.d.	n.d.	n.d.	n.d.	n.d.

	G250R	30	no	negative	undet.	n.d.	n.d.	n.d.	n.d.	n.d.
G251	G251C	30	no	negative	undet.	n.d.	n.d.	n.d.	n.d.	n.d.
	G251R	30	no	negative	undet.	n.d.	n.d.	n.d.	n.d.	n.d.
G252	G252C	30	no	negative	undet.	n.d.	n.d.	n.d.	n.d.	n.d.
	G252R	30	no	negative	undet.	n.d.	n.d.	n.d.	n.d.	n.d.
G253	G253C	30	no	negative	undet.	n.d.	n.d.	n.d.	n.d.	n.d.
	G253R	30	no	negative	undet.	n.d.	n.d.	n.d.	n.d.	n.d.
G254	G254C	30	no	negative	undet.	n.d.	n.d.	n.d.	n.d.	n.d.
	G254R	30	no	negative	undet.	n.d.	n.d.	n.d.	n.d.	n.d.
G255	G255C	30	no	negative	undet.	n.d.	n.d.	n.d.	n.d.	n.d.
	G255R	30	no	negative	undet.	n.d.	n.d.	n.d.	n.d.	n.d.
G256	G256C	30	no	negative	undet.	n.d.	n.d.	n.d.	n.d.	n.d.
	G256R	30	no	negative	undet.	n.d.	n.d.	n.d.	n.d.	n.d.
G257	G257C	30	yes	negative	undet.	n.d.	n.d.	n.d.	n.d.	n.d.
	G257R	30	yes	negative	undet.	n.d.	n.d.	n.d.	n.d.	n.d.
G258	G258C	30	yes	negative	undet.	n.d.	n.d.	n.d.	n.d.	n.d.
	G258R	30	yes	negative	undet.	n.d.	n.d.	n.d.	n.d.	n.d.
G259	G259C	30	no	negative	undet.	n.d.	n.d.	n.d.	n.d.	n.d.
	G259R	30	no	negative	undet.	n.d.	n.d.	n.d.	n.d.	n.d.
G260	G260C	30	yes	negative	undet.	n.d.	n.d.	n.d.	n.d.	n.d.
	G260R	30	yes	negative	undet.	n.d.	n.d.	n.d.	n.d.	n.d.
R1	R1C	9	no	negative	undet.	n.d.	n.d.	n.d.	n.d.	n.d.
	R1R	9	no	negative	undet.	n.d.	n.d.	n.d.	n.d.	n.d.
R2	R2C	9	no	negative	undet.	n.d.	n.d.	n.d.	n.d.	n.d.
	R2R	9	no	negative	undet.	n.d.	n.d.	n.d.	n.d.	n.d.
R3	R3C	9	no	negative	undet.	n.d.	n.d.	n.d.	n.d.	n.d.
	R3R	9	no	negative	undet.	n.d.	n.d.	n.d.	n.d.	n.d.
R4	R4C	9	no	negative	undet.	n.d.	n.d.	n.d.	n.d.	n.d.
	R4R	9	no	negative	undet.	n.d.	n.d.	n.d.	n.d.	n.d.
R5	R5C	9	no	negative	undet.	n.d.	n.d.	n.d.	n.d.	n.d.
	R5R	9	no	negative	undet.	n.d.	n.d.	n.d.	n.d.	n.d.

R6	R6C	9	no	negative	undet.	n.d.	n.d.	n.d.	n.d.	n.d.
	R6R	9	no	negative	undet.	n.d.	n.d.	n.d.	n.d.	n.d.
R7	R7C	10	no	negative	undet.	n.d.	n.d.	n.d.	n.d.	n.d.
	R7R	10	no	negative	undet.	n.d.	n.d.	n.d.	n.d.	n.d.
R8	R8C	11	no	negative	undet.	n.d.	n.d.	n.d.	n.d.	n.d.
	R8R	11	no	negative	undet.	n.d.	n.d.	n.d.	n.d.	n.d.
R9	R9C	11	no	negative	undet.	n.d.	n.d.	n.d.	n.d.	n.d.
	R9R	11	no	negative	undet.	n.d.	n.d.	n.d.	n.d.	n.d.
R10	R10C	11	no	negative	undet.	n.d.	n.d.	n.d.	n.d.	n.d.
	R10R	11	no	negative	undet.	n.d.	n.d.	n.d.	n.d.	n.d.
R11	R11C	12	no	negative	undet.	n.d.	n.d.	n.d.	n.d.	n.d.
	R11R	12	no	negative	undet.	n.d.	n.d.	n.d.	n.d.	n.d.
R12	R12C_right	12	yes	negative	undet.	n.d.	n.d.	n.d.	n.d.	n.d.
	R12R	12	yes	negative	undet.	n.d.	n.d.	n.d.	n.d.	n.d.
	R12C_left	12	yes	negative	undet.	n.d.	n.d.	n.d.	n.d.	n.d.
R13	R13C	12	no	negative	undet.	n.d.	n.d.	n.d.	n.d.	n.d.
	R13R	12	no	negative	undet.	n.d.	n.d.	n.d.	n.d.	n.d.
R14	R14C	12	no	negative	undet.	n.d.	n.d.	n.d.	n.d.	n.d.
	R14R	12	no	negative	undet.	n.d.	n.d.	n.d.	n.d.	n.d.
R15	R15C_right	13	yes	negative	undet.	n.d.	n.d.	n.d.	n.d.	n.d.
	R15R	13	yes	negative	undet.	n.d.	n.d.	n.d.	n.d.	n.d.
	R15C_left	13	yes	negative	undet.	n.d.	n.d.	n.d.	n.d.	n.d.
R16	R16C	13	no	negative	undet.	n.d.	n.d.	n.d.	n.d.	n.d.
	R16R	13	no	negative	undet.	n.d.	n.d.	n.d.	n.d.	n.d.
R17	R17C	13	no	negative	undet.	n.d.	n.d.	n.d.	n.d.	n.d.
	R17R	13	no	negative	undet.	n.d.	n.d.	n.d.	n.d.	n.d.
R18	R18C	14	no	negative	undet.	n.d.	n.d.	n.d.	n.d.	n.d.
	R18R	14	no	negative	undet.	n.d.	n.d.	n.d.	n.d.	n.d.
R19	R19C	14	no	negative	undet.	n.d.	n.d.	n.d.	n.d.	n.d.
	R19R	14	no	negative	undet.	n.d.	n.d.	n.d.	n.d.	n.d.
R20	R20C	14	no	negative	undet.	n.d.	n.d.	n.d.	n.d.	n.d.

	R20R	14	no	negative	undet.	n.d.	n.d.	n.d.	n.d.	n.d.
R21	R21C	14	no	negative	undet.	n.d.	n.d.	n.d.	n.d.	n.d.
	R21R	14	no	negative	undet.	n.d.	n.d.	n.d.	n.d.	n.d.
R22	R22C	14	yes	negative	undet.	n.d.	n.d.	n.d.	n.d.	n.d.
	R22R	14	yes	negative	undet.	n.d.	n.d.	n.d.	n.d.	n.d.
R23	R23C	14	no	negative	undet.	n.d.	n.d.	n.d.	n.d.	n.d.
	R23R	14	no	negative	undet.	n.d.	n.d.	n.d.	n.d.	n.d.
R24	R24C	14	no	negative	undet.	n.d.	n.d.	n.d.	n.d.	n.d.
	R24R	14	no	negative	undet.	n.d.	n.d.	n.d.	n.d.	n.d.
R25	R25C	14	no	negative	undet.	n.d.	n.d.	n.d.	n.d.	n.d.
	R25R	14	no	negative	undet.	n.d.	n.d.	n.d.	n.d.	n.d.
R26	R26C	14	no	negative	undet.	n.d.	n.d.	n.d.	n.d.	n.d.
	R26R	14	no	negative	undet.	n.d.	n.d.	n.d.	n.d.	n.d.
R27	R27C	14	no	negative	undet.	n.d.	n.d.	n.d.	n.d.	n.d.
	R27R	14	no	negative	undet.	n.d.	n.d.	n.d.	n.d.	n.d.
R28	R28C	15	no	negative	undet.	n.d.	n.d.	n.d.	n.d.	n.d.
	R28R	15	no	negative	undet.	n.d.	n.d.	n.d.	n.d.	n.d.
R29	R29C	15	no	negative	undet.	n.d.	n.d.	n.d.	n.d.	n.d.
	R29R	15	no	negative	undet.	n.d.	n.d.	n.d.	n.d.	n.d.
R30	R30C	15	no	negative	undet.	n.d.	n.d.	n.d.	n.d.	n.d.
	R30R	15	no	negative	undet.	n.d.	n.d.	n.d.	n.d.	n.d.
R31	R31C_right	16	yes	negative	undet.	n.d.	n.d.	n.d.	n.d.	n.d.
	R31R	16	yes	negative	undet.	n.d.	n.d.	n.d.	n.d.	n.d.
	R31C_left	16	yes	negative	undet.	n.d.	n.d.	n.d.	n.d.	n.d.
R32	R32C	16	no	negative	undet.	n.d.	n.d.	n.d.	n.d.	n.d.
	R32R	16	no	negative	undet.	n.d.	n.d.	n.d.	n.d.	n.d.
R33	R33C_right	17	yes	negative	undet.	n.d.	n.d.	n.d.	n.d.	n.d.
	R33R	17	yes	negative	undet.	n.d.	n.d.	n.d.	n.d.	n.d.
	R33C_left	17	yes	negative	undet.	n.d.	n.d.	n.d.	n.d.	n.d.
R34	R34C	17	no	negative	undet.	n.d.	n.d.	n.d.	n.d.	n.d.
	R34R	17	no	negative	undet.	n.d.	n.d.	n.d.	n.d.	n.d.

R35	R35C	18	no	negative	undet.	n.d.	n.d.	n.d.	n.d.	n.d.
	R35R	18	no	negative	undet.	n.d.	n.d.	n.d.	n.d.	n.d.
R36	R36C	19	no	negative	undet.	n.d.	n.d.	n.d.	n.d.	n.d.
	R36R	19	no	negative	undet.	n.d.	n.d.	n.d.	n.d.	n.d.
R37	R37C	19	no	negative	undet.	n.d.	n.d.	n.d.	n.d.	n.d.
	R37R	19	no	negative	undet.	n.d.	n.d.	n.d.	n.d.	n.d.
R38	R38C	19	no	negative	undet.	n.d.	n.d.	n.d.	n.d.	n.d.
	R38R	19	no	negative	undet.	n.d.	n.d.	n.d.	n.d.	n.d.
R39	R39C	19	no	negative	undet.	n.d.	n.d.	n.d.	n.d.	n.d.
	R39R	19	no	negative	undet.	n.d.	n.d.	n.d.	n.d.	n.d.
R40	R40C	19	no	negative	undet.	n.d.	n.d.	n.d.	n.d.	n.d.
	R40R	19	no	negative	undet.	n.d.	n.d.	n.d.	n.d.	n.d.
R41	R41C	20	no	negative	undet.	n.d.	n.d.	n.d.	n.d.	n.d.
	R41R	20	no	negative	undet.	n.d.	n.d.	n.d.	n.d.	n.d.
R42	R42C	20	no	negative	undet.	n.d.	n.d.	n.d.	n.d.	n.d.
	R42R	20	no	negative	undet.	n.d.	n.d.	n.d.	n.d.	n.d.
R43	R43C_right	21	yes	negative	undet.	n.d.	n.d.	n.d.	n.d.	n.d.
	R43R	21	yes	negative	undet.	n.d.	n.d.	n.d.	n.d.	n.d.
	R43C_left	21	yes	negative	undet.	n.d.	n.d.	n.d.	n.d.	n.d.
R44	R44C	21	no	negative	undet.	n.d.	n.d.	n.d.	n.d.	n.d.
	R44R	21	no	negative	undet.	n.d.	n.d.	n.d.	n.d.	n.d.
R45	R45C_right	21	yes	negative	undet.	n.d.	n.d.	n.d.	n.d.	n.d.
	R45R	21	yes	negative	undet.	n.d.	n.d.	n.d.	n.d.	n.d.
	R45C_left	21	yes	negative	undet.	n.d.	n.d.	n.d.	n.d.	n.d.
R46	R46C	21	yes	negative	undet.	n.d.	n.d.	n.d.	n.d.	n.d.
	R46R	21	yes	negative	undet.	n.d.	n.d.	n.d.	n.d.	n.d.
R47	R47C	21	no	negative	undet.	n.d.	n.d.	n.d.	n.d.	n.d.
	R47R	21	no	negative	undet.	n.d.	n.d.	n.d.	n.d.	n.d.
R48	R48C_right	22	yes	negative	undet.	n.d.	n.d.	n.d.	n.d.	n.d.
	R48R	22	yes	negative	undet.	n.d.	n.d.	n.d.	n.d.	n.d.
	R48C_left	22	yes	negative	undet.	n.d.	n.d.	n.d.	n.d.	n.d.

R49	R49C	22	no	negative	undet.	n.d.	n.d.	n.d.	n.d.	n.d.
	R49R	22	no	negative	undet.	n.d.	n.d.	n.d.	n.d.	n.d.
R50	R50C	22	no	negative	undet.	n.d.	n.d.	n.d.	n.d.	n.d.
	R50R	22	no	negative	undet.	n.d.	n.d.	n.d.	n.d.	n.d.
R51	R51C	22	no	negative	undet.	n.d.	n.d.	n.d.	n.d.	n.d.
	R51R	22	no	negative	undet.	n.d.	n.d.	n.d.	n.d.	n.d.
R52	R52C	23	yes	negative	undet.	n.d.	n.d.	n.d.	n.d.	n.d.
	R52R	23	yes	negative	undet.	n.d.	n.d.	n.d.	n.d.	n.d.
R53	R53C	23	yes	negative	undet.	n.d.	n.d.	n.d.	n.d.	n.d.
	R53R	23	yes	negative	undet.	n.d.	n.d.	n.d.	n.d.	n.d.
R54	R54C	24	yes	negative	undet.	n.d.	n.d.	n.d.	n.d.	n.d.
	R54R	24	yes	negative	undet.	n.d.	n.d.	n.d.	n.d.	n.d.
R55	R55C	24	yes	negative	undet.	n.d.	n.d.	n.d.	n.d.	n.d.
	R55R	24	yes	negative	undet.	n.d.	n.d.	n.d.	n.d.	n.d.
R56	R56C	24	no	negative	undet.	n.d.	n.d.	n.d.	n.d.	n.d.
	R56R	24	no	negative	undet.	n.d.	n.d.	n.d.	n.d.	n.d.
R57	R57C	24	no	negative	undet.	n.d.	n.d.	n.d.	n.d.	n.d.
	R57R	24	no	negative	undet.	n.d.	n.d.	n.d.	n.d.	n.d.
R58	R58C	24	yes	negative	undet.	n.d.	n.d.	n.d.	n.d.	n.d.
	R58R	24	yes	negative	undet.	n.d.	n.d.	n.d.	n.d.	n.d.
R59	R59C_right	24	yes	negative	undet.	n.d.	n.d.	n.d.	n.d.	n.d.
	R59R	24	yes	negative	undet.	n.d.	n.d.	n.d.	n.d.	n.d.
	R59C_left	24	yes	positive	32.66	99.62% *C. psittaci* (CP003790.1)	n.d.	positive	n.i.	n.d.
R60	R60C_right	25	yes	negative	undet.	n.d.	n.d.	n.d.	n.d.	n.d.
	R60R	25	yes	negative	undet.	n.d.	n.d.	n.d.	n.d.	n.d.
	R60C_left	25	yes	negative	undet.	n.d.	n.d.	n.d.	n.d.	n.d.
R61	R61C	26	no	negative	undet.	n.d.	n.d.	n.d.	n.d.	n.d.
	R61R	26	no	negative	undet.	n.d.	n.d.	n.d.	n.d.	n.d.
R62	R62C	26	yes	negative	undet.	n.d.	n.d.	n.d.	n.d.	n.d.
	R62R	26	yes	negative	undet.	n.d.	n.d.	n.d.	n.d.	n.d.

R63	R63C	26	no	negative	undet.	n.d.	n.d.	n.d.	n.d.	n.d.
	R63R	26	no	negative	undet.	n.d.	n.d.	n.d.	n.d.	n.d.
R64	R64C_right	26	yes	negative	undet.	n.d.	n.d.	n.d.	n.d.	n.d.
	R64R	26	yes	negative	undet.	n.d.	n.d.	n.d.	n.d.	n.d.
	R64C_left	26	yes	negative	undet.	n.d.	n.d.	n.d.	n.d.	n.d.
R65	R65C	26	no	negative	undet.	n.d.	n.d.	n.d.	n.d.	n.d.
	R65R	26	no	negative	undet.	n.d.	n.d.	n.d.	n.d.	n.d.
R66	R66C	26	no	negative	undet.	n.d.	n.d.	n.d.	n.d.	n.d.
	R66R	26	no	negative	undet.	n.d.	n.d.	n.d.	n.d.	n.d.
R67	R67C	26	yes	negative	undet.	n.d.	n.d.	n.d.	n.d.	n.d.
	R67R	26	yes	negative	undet.	n.d.	n.d.	n.d.	n.d.	n.d.
R68	R68C	26	yes	positive	32.32	99.43% *C. psittaci* (CP003790.1)	n.d.	positive	97.3% *C. psittaci* (CP003790.1)	n.d.
	R68R	26	yes	negative	undet.	n.d.	n.d.	n.d.	n.d.	n.d.
R69	R69C_right	26	yes	negative	undet.	n.d.	n.d.	n.d.	n.d.	n.d.
	R69R	26	yes	negative	undet.	n.d.	n.d.	n.d.	n.d.	n.d.
	R69C_left	26	yes	negative	undet.	n.d.	n.d.	n.d.	n.d.	n.d.
R70	R70C	27	yes	negative	undet.	n.d.	n.d.	n.d.	n.d.	n.d.
	R70R	27	yes	negative	undet.	n.d.	n.d.	n.d.	n.d.	n.d.
R71	R71C	29	yes	negative	undet.	n.d.	n.d.	n.d.	n.d.	n.d.
	R71R	29	yes	negative	undet.	n.d.	n.d.	n.d.	n.d.	n.d.
R72	R72C	30	no	negative	undet.	n.d.	n.d.	n.d.	n.d.	n.d.
	R72R	30	no	negative	undet.	n.d.	n.d.	n.d.	n.d.	n.d.
R73	R73C	30	yes	negative	undet.	n.d.	n.d.	n.d.	n.d.	n.d.
	R73R	30	yes	negative	undet.	n.d.	n.d.	n.d.	n.d.	n.d.
R74	R74C	30	no	negative	undet.	n.d.	n.d.	n.d.	n.d.	n.d.
	R74R	30	no	negative	undet.	n.d.	n.d.	n.d.	n.d.	n.d.
R75	R75C	30	yes	negative	undet.	n.d.	n.d.	n.d.	n.d.	n.d.
	R75R	30	yes	negative	undet.	n.d.	n.d.	n.d.	n.d.	n.d.
R76	R76C	31	yes	negative	undet.	n.d.	n.d.	n.d.	n.d.	n.d.
	R76R	31	yes	negative	undet.	n.d.	n.d.	n.d.	n.d.	n.d.

R77	R77C	32	yes	negative	undet.	n.d.	n.d.	n.d.	n.d.	n.d.
	R77R	32	yes	negative	undet.	n.d.	n.d.	n.d.	n.d.	n.d.
R78	R78C	33	no	negative	undet.	n.d.	n.d.	n.d.	n.d.	n.d.
	R78R	33	no	negative	undet.	n.d.	n.d.	n.d.	n.d.	n.d.
R79	R79C	34	no	negative	undet.	n.d.	n.d.	n.d.	n.d.	n.d.
	R79R	34	no	negative	undet.	n.d.	n.d.	n.d.	n.d.	n.d.
R80	R80C	35	no	negative	undet.	n.d.	n.d.	n.d.	n.d.	n.d.
	R80R	35	no	negative	undet.	n.d.	n.d.	n.d.	n.d.	n.d.
R81	R81C	36	data not available	negative	undet.	n.d.	n.d.	n.d.	n.d.	n.d.
	R81R	36	data not available	negative	undet.	n.d.	n.d.	n.d.	n.d.	n.d.
R82	R82C	37	yes	negative	undet.	n.d.	n.d.	n.d.	n.d.	n.d.
	R82R	37	yes	negative	undet.	n.d.	n.d.	n.d.	n.d.	n.d.
R83	R83C	38	yes	negative	undet.	n.d.	n.d.	n.d.	n.d.	n.d.
	R83R	38	yes	negative	undet.	n.d.	n.d.	n.d.	n.d.	n.d.
R84	R84C	39	no	negative	undet.	n.d.	n.d.	n.d.	n.d.	n.d.
	R84R	39	no	negative	undet.	n.d.	n.d.	n.d.	n.d.	n.d.
R85	R85C	40	no	negative	undet.	n.d.	n.d.	n.d.	n.d.	n.d.
	R85R	40	no	negative	undet.	n.d.	n.d.	n.d.	n.d.	n.d.
R86	R86C	41	data not available	negative	undet.	n.d.	n.d.	n.d.	n.d.	n.d.
	R86R	41	data not available	negative	undet.	n.d.	n.d.	n.d.	n.d.	n.d.
R87	R87C	42	data not available	negative	undet.	n.d.	n.d.	n.d.	n.d.	n.d.
	R87R	42	data not available	negative	undet.	n.d.	n.d.	n.d.	n.d.	n.d.
R88	R88C	43	no	negative	undet.	n.d.	n.d.	n.d.	n.d.	n.d.
	R88R	43	no	negative	undet.	n.d.	n.d.	n.d.	n.d.	n.d.
R89	R89C	44	yes	negative	undet.	n.d.	n.d.	n.d.	n.d.	n.d.

	R89R	44	yes	negative	undet.	n.d.	n.d.	n.d.	n.d.	n.d.
R90	R90C	45	no	negative	undet.	n.d.	n.d.	n.d.	n.d.	n.d.
	R90R	45	no	negative	undet.	n.d.	n.d.	n.d.	n.d.	n.d.
R91	R91C	46	no	negative	undet.	n.d.	n.d.	n.d.	n.d.	n.d.
	R91R	46	no	negative	undet.	n.d.	n.d.	n.d.	n.d.	n.d.
R92	R92C	47	no	negative	undet.	n.d.	n.d.	n.d.	n.d.	n.d.
	R92R	47	no	negative	undet.	n.d.	n.d.	n.d.	n.d.	n.d.
R93	R93C	48	no	negative	undet.	n.d.	n.d.	n.d.	n.d.	n.d.
	R93R	48	no	negative	undet.	n.d.	n.d.	n.d.	n.d.	n.d.
R94	R94C	49	no	negative	undet.	n.d.	n.d.	n.d.	n.d.	n.d.
	R94R	49	no	negative	undet.	n.d.	n.d.	n.d.	n.d.	n.d.
R95	R95C	50	no	negative	undet.	n.d.	n.d.	n.d.	n.d.	n.d.
	R95R	50	no	negative	undet.	n.d.	n.d.	n.d.	n.d.	n.d.
R96	R96C	51	no	negative	undet.	n.d.	n.d.	n.d.	n.d.	n.d.
	R96R	51	no	negative	undet.	n.d.	n.d.	n.d.	n.d.	n.d.
R97	R97C	52	yes	negative	undet.	n.d.	n.d.	n.d.	n.d.	n.d.
	R97R	52	yes	negative	undet.	n.d.	n.d.	n.d.	n.d.	n.d.
R98	R98C	53	yes	negative	undet.	n.d.	n.d.	n.d.	n.d.	n.d.
	R98R	53	yes	negative	undet.	n.d.	n.d.	n.d.	n.d.	n.d.
R99	R99C	54	no	negative	undet.	n.d.	n.d.	n.d.	n.d.	n.d.
	R99R	54	no	negative	undet.	n.d.	n.d.	n.d.	n.d.	n.d.
R100	R100C	55	yes	negative	undet.	n.d.	n.d.	n.d.	n.d.	n.d.
	R100R	55	yes	negative	undet.	n.d.	n.d.	n.d.	n.d.	n.d.
R101	R101C	55	no	negative	undet.	n.d.	n.d.	n.d.	n.d.	n.d.
	R101R	55	no	negative	undet.	n.d.	n.d.	n.d.	n.d.	n.d.
R102	R102C	56	yes	negative	undet.	n.d.	n.d.	n.d.	n.d.	n.d.
	R102R	56	yes	negative	undet.	n.d.	n.d.	n.d.	n.d.	n.d.
R103	R103C	57	no	positive	37.58	n.i.	negative	negative	n.d.	n.d.
	R103R	57	no	negative	undet.	n.d.	n.d.	n.d.	n.d.	n.d.
R104	R104C	58	no	negative	undet.	n.d.	n.d.	n.d.	n.d.	n.d.
	R104R	58	no	negative	undet.	n.d.	n.d.	n.d.	n.d.	n.d.

R105	R105C	59	no	negative	undet.	n.d.	n.d.	n.d.	n.d.	n.d.
	R105R	59	no	negative	undet.	n.d.	n.d.	n.d.	n.d.	n.d.
R106	R106C	60	yes	negative	undet.	n.d.	n.d.	n.d.	n.d.	n.d.
	R106R	60	yes	negative	undet.	n.d.	n.d.	n.d.	n.d.	n.d.
R107	R107C	61	no	negative	undet.	n.d.	n.d.	n.d.	n.d.	n.d.
	R107R	61	no	negative	undet.	n.d.	n.d.	n.d.	n.d.	n.d.
R108	R108C	62	data not available	negative	undet.	n.d.	n.d.	n.d.	n.d.	n.d.
	R108R	62	data not available	negative	undet.	n.d.	n.d.	n.d.	n.d.	n.d.
R109	R109C	63	no	negative	undet.	n.d.	n.d.	n.d.	n.d.	n.d.
	R109R	63	no	negative	undet.	n.d.	n.d.	n.d.	n.d.	n.d.
R110	R110C	64	no	negative	undet.	n.d.	n.d.	n.d.	n.d.	n.d.
	R110R	64	no	negative	undet.	n.d.	n.d.	n.d.	n.d.	n.d.

[A]G: guinea pig: R: rabbit
[B]C: conjunctival swab; R: rectal swab
[1]limited amount of DNA
n.d.: not done
n.i.: not identified

Supplementary Table 2: Details on sample identity, diagnostics performed, and subsequent results of each conjunctival composite swab sampled in the Netherlands (qPCR: real-time PCR; PCR: conventional PCR).

**Sample ID**	**Breeder ID**	**Number of guinea pigs per swab**	**Presence of clinical signs (ocular or nasal discharge, ocular pathologies)**	**23S *Chlamydiaceae* qPCR**		**VD4 *C. caviae ompA* - Sequencing result**	***C. caviae* complete *ompA* gene PCR -Sequencing result**
				**Result**	**Mean Ct value**		
1	1	5	no	negative	undet.	n.d.	n.d.
2	1	5	no	negative	undet.	n.d.	n.d.
3	1	5	no	negative	undet.	n.d.	n.d.
4	1	5	no	negative	undet.	n.d.	n.d.
5	1	4	no	negative	undet.	n.d.	n.d.
6	1	1	yes	negative	undet.	n.d.	n.d.
7	2	5	no	negative	undet.	n.d.	n.d.
8	2	3	no	negative	undet.	n.d.	n.d.
9	3	5	no	negative	undet.	n.d.	n.d.
10	3	5	no	negative	undet.	n.d.	n.d.
11	3	4	no	negative	undet.	n.d.	n.d.
12	3	1	yes	negative	undet.	n.d.	n.d.
13	4	5	no	negative	undet.	n.d.	n.d.
14	4	5	no	negative	undet.	n.d.	n.d.
15	4	2	no	negative	undet.	n.d.	n.d.
16	5	5	no	negative	undet.	n.d.	n.d.
17	5	5	no	negative	undet.	n.d.	n.d.
18	5	5	no	negative	undet.	n.d.	n.d.
19	5	5	no	negative	undet.	n.d.	n.d.
20	5	5	no	positive	31.1	*C. caviae* (KY777661)	n.i.
21	5	3	no	negative	undet.	n.d.	n.d.
22	6	5	no	negative	undet.	n.d.	n.d.
23	6	5	no	negative	undet.	n.d.	n.d.

24	6	4	no	negative	undet.	n.d.	n.d.
25	7	5	no	negative	undet.	n.d.	n.d.
26	7	5	no	negative	undet.	n.d.	n.d.
27	7	1	yes	negative	undet.	n.d.	n.d.
28	7	3	no	negative	undet.	n.d.	n.d.
29	8	5	no	negative	undet.	n.d.	n.d.
30	8	5	no	negative	undet.	n.d.	n.d.
31	8	5	no	negative	undet.	n.d.	n.d.
32	8	5	no	negative	undet.	n.d.	n.d.
33	9	5	no	negative	undet.	n.d.	n.d.
34	9	5	no	negative	undet.	n.d.	n.d.
35	9	5	no	negative	undet.	n.d.	n.d.
36	9	4	no	negative	undet.	n.d.	n.d.
37	10	5	no	negative	undet.	n.d.	n.d.
38	10	6	no	negative	undet.	n.d.	n.d.
39	10	5	no	negative	undet.	n.d.	n.d.
40	10	4	no	negative	undet.	n.d.	n.d.
41	10	1	yes	negative	undet.	n.d.	n.d.
42	11	1	yes	negative	undet.	n.d.	n.d.
43	11	5	no	negative	undet.	n.d.	n.d.
44	11	5	no	negative	undet.	n.d.	n.d.
45	11	5	no	negative	undet.	n.d.	n.d.
46	12	5	no	negative	undet.	n.d.	n.d.
47	12	5	no	negative	undet.	n.d.	n.d.
48	12	5	no	negative	undet.	n.d.	n.d.
49	13	5	no	negative	undet.	n.d.	n.d.
50	13	5	no	positive	25.5	*C. caviae* (KY777661)	*C. caviae* (KY777661)
51	13	3	no	positive	31.9	*C. caviae* (KY777661)	n.i.
52	13	5	no	positive	24.6	*C. caviae* (KY777661)	*C. caviae* (KY777661)
53	13	4	no	questionable	36.3	*C. caviae* (KY777661)	n.i.

54	13	2	yes	negative	undet.	n.d.	n.d.
55	13	5	no	positive	26.1	*C. caviae* (KY777661)	*C. caviae* (KY777661)
56	13	5	no	negative	undet.	n.d.	n.d.
57	13	5	no	negative	undet.	n.d.	n.d.
58	13	5	no	positive	31.1	*C. caviae* (KY777661)	n.i.
59	13	5	no	negative	undet.	n.d.	n.d.
60	13	5	no	positive	29.5	*C. caviae* (KY777661)	n.i.
61	13	4	no	negative	undet.	n.d.	n.d.
62	13	5	no	positive	33.5	n.i.	n.i.
63	13	5	no	negative	undet.	n.d.	n.d.
64	13	5	no	negative	undet.	n.d.	n.d.
65	13	4	no	negative	undet.	n.d.	n.d.
66	13	4	no	negative	undet.	n.d.	n.d.
67	13	5	yes	positive	27	PCR positive, but sequence undet.	n.i.
68	13	1	yes	negative	undet.	n.d.	n.d.
69	14	4	no	negative	undet.	n.d.	n.d.
70	14	4	no	negative	undet.	n.d.	n.d.
71	15	5	no	negative	undet.	n.d.	n.d.
72	15	5	no	negative	undet.	n.d.	n.d.
73	15	5	no	negative	undet.	n.d.	n.d.
74	15	5	no	negative	undet.	n.d.	n.d.
75	15	5	no	negative	undet.	n.d.	n.d.
76	15	5	no	negative	undet.	n.d.	n.d.
77	15	5	no	negative	undet.	n.d.	n.d.
78	15	5	no	negative	undet.	n.d.	n.d.
79	15	1	no	negative	undet.	n.d.	n.d.
80	15	1	yes	negative	undet.	n.d.	n.d.
81	16	5	no	negative	undet.	n.d.	n.d.
82	16	6	no	negative	undet.	n.d.	n.d.

83	17	5	no	negative	undet.	n.d.	n.d.
84	17	5	no	negative	undet.	n.d.	n.d.
85	17	5	no	negative	undet.	n.d.	n.d.
86	17	5	no	negative	undet.	n.d.	n.d.
87	17	5	no	negative	undet.	n.d.	n.d.
88	17	5	no	negative	undet.	n.d.	n.d.
89	17	5	no	negative	undet.	n.d.	n.d.
90	17	5	no	negative	undet.	n.d.	n.d.
91	17	4	no	negative	undet.	n.d.	n.d.
92	18	5	no	negative	undet.	n.d.	n.d.
93	19	5	no	negative	undet.	n.d.	n.d.
94	19	5	no	negative	undet.	n.d.	n.d.
95	19	5	no	negative	undet.	n.d.	n.d.
96	19	5	no	negative	undet.	n.d.	n.d.
97	19	5	no	negative	undet.	n.d.	n.d.
98	19	3	no	questionable	37.7	n.i.	n.i.
99	19	3	no	negative	undet.	n.d.	n.d.
100	20	5	no	negative	undet.	n.d.	n.d.
101	20	5	no	negative	undet.	n.d.	n.d.
102	20	5	no	negative	undet.	n.d.	n.d.
103	20	5	no	negative	undet.	n.d.	n.d.
104	20	4	no	negative	undet.	n.d.	n.d.
105	21	5	no	negative	undet.	n.d.	n.d.
106	21	5	no	negative	undet.	n.d.	n.d.
107	21	5	no	negative	undet.	n.d.	n.d.
108	21	4	no	negative	undet.	n.d.	n.d.
109	22	5	no	negative	undet.	n.d.	n.d.
110	22	5	no	negative	undet.	n.d.	n.d.
111	22	2	no	negative	undet.	n.d.	n.d.
112	23	5	no	negative	undet.	n.d.	n.d.

113	23	4	no	negative	undet.	n.d.	n.d.
114	23	5	no	negative	undet.	n.d.	n.d.
115	24	5	no	negative	undet.	n.d.	n.d.
116	24	4	no	negative	undet.	n.d.	n.d.
117	25	5	no	negative	undet.	n.d.	n.d.
118	25	5	no	negative	undet.	n.d.	n.d.
119	25	4	no	negative	undet.	n.d.	n.d.
120	25	1	yes	negative	undet.	n.d.	n.d.
121	26	5	no	negative	undet.	n.d.	n.d.
122	26	5	no	negative	undet.	n.d.	n.d.
123	26	5	no	negative	undet.	n.d.	n.d.
124	26	5	no	negative	undet.	n.d.	n.d.
125	26	5	no	negative	undet.	n.d.	n.d.
126	26	5	no	negative	undet.	n.d.	n.d.
127	26	5	no	negative	undet.	n.d.	n.d.
128	26	5	no	negative	undet.	n.d.	n.d.
129	26	5	no	negative	undet.	n.d.	n.d.
130	27	5	no	negative	undet.	n.d.	n.d.
131	27	5	no	negative	undet.	n.d.	n.d.
132	27	5	no	negative	undet.	n.d.	n.d.
133	27	1	yes	negative	undet.	n.d.	n.d.
134	27	5	no	negative	undet.	n.d.	n.d.
135	27	4	no	negative	undet.	n.d.	n.d.
136	27	6	no	negative	undet.	n.d.	n.d.
137	28	4	no	negative	undet.	n.d.	n.d.
138	28	4	no	questionable	37.2	n.i.	n.i.
139	30	4	no	negative	undet.	n.d.	n.d.
140	30	4	no	negative	undet.	n.d.	n.d.
141	30	4	no	negative	undet.	n.d.	n.d.
142	30	4	no	negative	undet.	n.d.	n.d.

143	30	4	no	negative	undet.	n.d.	n.d.
144	30	4	no	negative	undet.	n.d.	n.d.
145	30	4	no	negative	undet.	n.d.	n.d.
146	30	4	no	negative	undet.	n.d.	n.d.
147	30	1	yes	negative	undet.	n.d.	n.d.
148	31	2	no	negative	undet.	n.d.	n.d.
149	31	4	no	negative	undet.	n.d.	n.d.
150	32	5	no	negative	undet.	n.d.	n.d.
151	32	5	no	negative	undet.	n.d.	n.d.
152	32	5	no	negative	undet.	n.d.	n.d.
153	32	5	no	negative	undet.	n.d.	n.d.
154	32	5	no	negative	undet.	n.d.	n.d.
155	32	5	no	negative	undet.	n.d.	n.d.
156	32	3	no	negative	undet.	n.d.	n.d.
157	32	4	yes	negative	undet.	n.d.	n.d.
158	33	5	no	negative	undet.	n.d.	n.d.
159	33	5	no	negative	undet.	n.d.	n.d.
160	33	5	no	negative	undet.	n.d.	n.d.
161	33	5	no	negative	undet.	n.d.	n.d.
162	33	5	no	negative	undet.	n.d.	n.d.
163	33	5	no	negative	undet.	n.d.	n.d.
164	33	5	no	negative	undet.	n.d.	n.d.
165	33	5	no	negative	undet.	n.d.	n.d.
166	33	2	no	negative	undet.	n.d.	n.d.
167	34	5	no	negative	undet.	n.d.	n.d.
168	34	5	no	negative	undet.	n.d.	n.d.
169	34	5	no	negative	undet.	n.d.	n.d.
170	34	5	no	negative	undet.	n.d.	n.d.
171	34	5	no	negative	undet.	n.d.	n.d.
172	34	5	no	negative	undet.	n.d.	n.d.

173	34	1	yes	negative	undet.	n.d.	n.d.
174	35	5	no	negative	undet.	n.d.	n.d.
175	35	4	no	negative	undet.	n.d.	n.d.
176	35	1	yes	negative	undet.	n.d.	n.d.
177	35	5	no	negative	undet.	n.d.	n.d.
178	35	4	no	negative	undet.	n.d.	n.d.
179	36	6	no	negative	undet.	n.d.	n.d.
180	36	4	no	negative	undet.	n.d.	n.d.
181	36	5	no	negative	undet.	n.d.	n.d.
182	37	5	no	negative	undet.	n.d.	n.d.
183	37	5	no	negative	undet.	n.d.	n.d.
184	37	5	no	negative	undet.	n.d.	n.d.
185	37	2	no	negative	undet.	n.d.	n.d.
186	37	4	no	negative	undet.	n.d.	n.d.
187	37	2	no	negative	undet.	n.d.	n.d.
188	38	3	no	positive	25	*C. caviae* (KY777661	*C. caviae* (KY777661)
189	38	3	no	questionable	36.1	PCR positive, but sequence undet.	n.i.
190	38	3	no	positive	24	PCR positive, but sequence undet.	*C. caviae* (KY777661)
191	38	3	no	positive	35.1	PCR positive, but sequence undet.	n.i.
192	38	5	no	positive	22	PCR positive, but sequence undet.	*C. caviae* (KY777661)
193	38	6	no	positive	22.8	PCR positive, but sequence undet.	*C. caviae* (KY777661)
194	38	4	no	positive	24	n.d.	*C. caviae* (KY777661)
195	38	2	no	positive	22.7	PCR positive, but sequence undet.	*C. caviae* (KY777661)
196	38	5	no	positive	23.7	PCR positive, but sequence undet.	*C. caviae* (KY777661)

197	38	4	no	positive	25.6	PCR positive, but sequence undet.	*C. caviae* (KY777661)
198	38	4	no	positive	25.7	*C. caviae* (KY777661)	n.i.
199	38	3	no	positive	25.4	PCR positive, but sequence undet.	*C. caviae* (KY777661)
200	38	6	yes	positive	22.4	*C. caviae* (KY777661)	*C. caviae* (KY777661)

undet.: undetermined
n.d.: not done
n.i.: not identified

Supplementary Table 3: Sequence comparison of specific genes of interest between strain NL_Conj_Li to strains 04DC41 and GPIC.

Putative function	Gene	04DC41	GPIC
sinC	CCA_00062	100.00%	99.87%
incA	CCA_00550	100.00%	99.63%
Cytotoxin	CCA_00558	99.91%	99.41%
polymorphic outer membrane protein	CCA_00208	100.00%	99.00%
polymorphic outer membrane protein	CCA_00274	100.00%	98.20%
polymorphic outer membrane protein	CCA_00275	100.00%	96.16%
polymorphic outer membrane protein	CCA_00276	100.00%	92.51%
polymorphic outer membrane protein	CCA_00277	100.00%	83.42%
polymorphic outer membrane protein	CCA_00278	-	93.61%
polymorphic outer membrane protein	CCA_00279	100.00%	98.74%
polymorphic outer membrane protein	CCA_00280	100.00%	99.02%
polymorphic outer membrane protein	CCA_00281	100.00%	99.52%
polymorphic outer membrane protein	CCA_00282	100.00%	98.74%
polymorphic outer membrane protein	CCA_00283	100.00%	98.78%

polymorphic outer membrane protein	CCA_00284	100.00%	98.81%
polymorphic outer membrane protein	CCA_00285	100.00%	99.23%
polymorphic outer membrane protein	CCA_00286	100.00%	99.37%
polymorphic outer membrane protein	CCA_00287	100.00%	99.33%
Inc proteins	CCA_0094	100.00%	99.49%
Inc proteins	CCA_00142	100.00%	100.00%
Inc proteins	CCA_00143	100.00%	100.00%
Inc proteins	CCA_00189	100.00%	100.00%
Inc proteins	CCA_00190	99.99%	99.74%
Inc proteins	CCA_00191	100.00%	100.00%
Inc proteins	CCA_00266	100.00%	99.72%
Inc proteins	CCA_00272	100.00%	98.81%
Inc proteins	CCA_00300	100.00%	99.78%
Inc proteins	CCA_00363	100.00%	99.84%
Inc proteins	CCA_00439	100.00%	99.65%
Inc proteins	CCA_00472	100.00%	99.92%

Inc proteins	CCA_00484	100.00%	99.78%
Inc proteins	CCA_00504	100.00%	98.93%
Inc proteins	CCA_00505	100.00%	96.98%
Inc proteins	CCA_00508	100.00%	99.91%
Inc proteins	CCA_00514	100.00%	98.40%
Inc proteins	CCA_00565	100.00%	99.63%
Inc proteins	CCA_00607	100.00%	99.86%
Inc proteins	CCA_00631	99.99%	99.70%
Inc proteins	CCA_00657	100.00%	98.95%
Inc proteins	CCA_00764	100.00%	99.92%
Inc proteins	CCA_00907	100.00%	99.73%
Inc proteins	CCA_00950	100.00%	99.78%
Inc proteins	CCA_00998	100.00%	99.75%
Inc proteins	CCA_01014	99.99%	99.17%
Inc proteins	CCA_01016	100.00%	99.02%
Plasmid	Complete plasmid	100.00%	99.81%

Supplementary Table 4: Content of each reaction mix and cycling protocols for the different real-time PCR (qPCR) and conventional PCR (PCR) methods used in this study.

Method	Reaction mix	Cycling protocol		
Chlamydiaceae 23S rRNA gene qPCR[2]	Total volume: 20 µl<br>-10 µl Taq-Man® Fast Universal PCR Master Mix (2) (Thermo Fisher Scientific)<br>-1000 nM primers Ch23S-F and Ch23S-R each<br>-200 nM probe Ch23S-p<br>-200 nM IPC probe<br>-1 µl IPC template<br>-0.2 µl Uracil-DNA Glycosylase (5U/µl)<br>-molecular grade water<br>-5 µl DNA sample	37 °C<br>95 °C<br>95 °C<br>60 °C	10 min<br>20 s<br>3 s<br>30 s	50x
Chlamydiaceae 23S rRNA gene qPCR[3]	Total volume: 25 µl<br>-1x TaqMan™ Fast Universal PCR Master Mix (Thermo Fisher Scientific)<br>-500 nM primers Ch23S-F and Ch23S-R each<br>-200 nM probe Ch23S-p<br>-100 nM eGFP-1-F and eGFP-10R each<br>-1 µl eGFP-HEX probe<br>-0.25 µl eGFP DNA template<br>-molecular grade water<br>-2.5 µl DNA sample	95 °C<br>95 °C<br>60 °C	20 s<br>3 s<br>30 s	45x
VD4 *C. caviae ompA* PCR[1]	Total volume: 25 µl<br>-12.5 µl SYBR® Select Master Mix (2X) (Thermo Fisher Scientific)<br>-500 nM primers CCVDF and CCVDR each<br>-molecular grade water<br>-5 µl DNA sample	95 °C<br>95 °C<br>60 °C<br><br>60-95 °C	3 min<br>5 s<br>60 s<br><br>Dissociation curve	40x
16S rRNA gene PCR[3]	Total volume: 50 µl<br>-1x AmpliTaq Gold™ 360 Master Mix (Thermo Fisher Scientific)<br>-300 nM primers 16S IGF and 16S IGR each	95 °C<br>95 °C<br>58 °C	10 min<br>30 s<br>30 s	40x

	-molecular grade water<br>-3 µl DNA sample	72 °C<br>72 °C	60 s<br>7 min	
C. psittaci-specific qPCR[3]	Total volume: 12.5 µl<br>-1x Path-ID™ qPCR Master Mix Kit (Thermo Fisher Scientific)<br>-900 nM primers CppsOMP1_For and CppsOMP1_Rev each<br>-200 nM probe CppsOMP1<br>-400 nM eGFP_For and eGFP_Rev each<br>-200 nM eGFP probe<br>-molecular grade water<br>-2.5 µl DNA sample	95 °C<br>95 °C<br>60 °C	10 min<br>15 s<br>60 s	45x
ompA Genotyping of *C. psittaci*[3]	Total volume: 50 µl<br>-1x AmpliTaq Gold™ 360 master mix (Thermo Fisher Scientific)<br>-200 nM primers ompA F (CTU) and ompA rev each<br>-molecular grade water<br>-3 µl DNA sample	95 °C<br>95 °C<br>49 °C<br>72 °C<br>72 °C	10 min<br>30 s<br>30 s<br>60 s<br>7 min	35x
C. caviae complete *ompA* gene PCR[2]	Total volume: 25 µl<br>-BioMix Red (Bioline)<br>-400 nM primers ompA_Fw1 and ompA_Rv1<br>-molecular grade water<br>-5 µl DNA sample	95 °C<br>94 °C<br>60 °C<br>72 °C	5 min<br>60 s<br>60 s<br>90 s	35x
C. caviae complete *ompA* gene PCR[3]	Total volume: 50 µl<br>-1x AmpliTaq Gold™ 360 master mix (Thermo Fisher Scientific)<br>-500 nM primers ompA_Fw1 and ompA_Rv1<br>-molecular grade water<br>-3 µl DNA sample	95 °C<br>95 °C<br>59 °C<br>72 °C	5 min<br>60 s<br>60 s<br>90 s	40x

[1]Method applied for Swiss and Dutch samples
[2]Method applied exclusively for the Dutch samples
[3]Method applied exclusively for the Swiss samples
